# Supplementary material for: A Humanized Yeast Phenomic Model of Deoxycytidine Kinase to Predict Genetic Buffering of Nucleoside Analog Cytotoxicity
Source: Genes (Basel). 2019 Sep 30;10(10):770. doi: 10.3390/genes10100770 (PMC6826991; doi:10.3390/genes10100770)
Supplement: Supplementary file 1 [file genes-10-00770-s001.zip › Additional_Files/Additional_File7_GOTermHeatmaps.zip!/Additional_File7_GOTermHeatmaps/organelle_membrane.pdf]

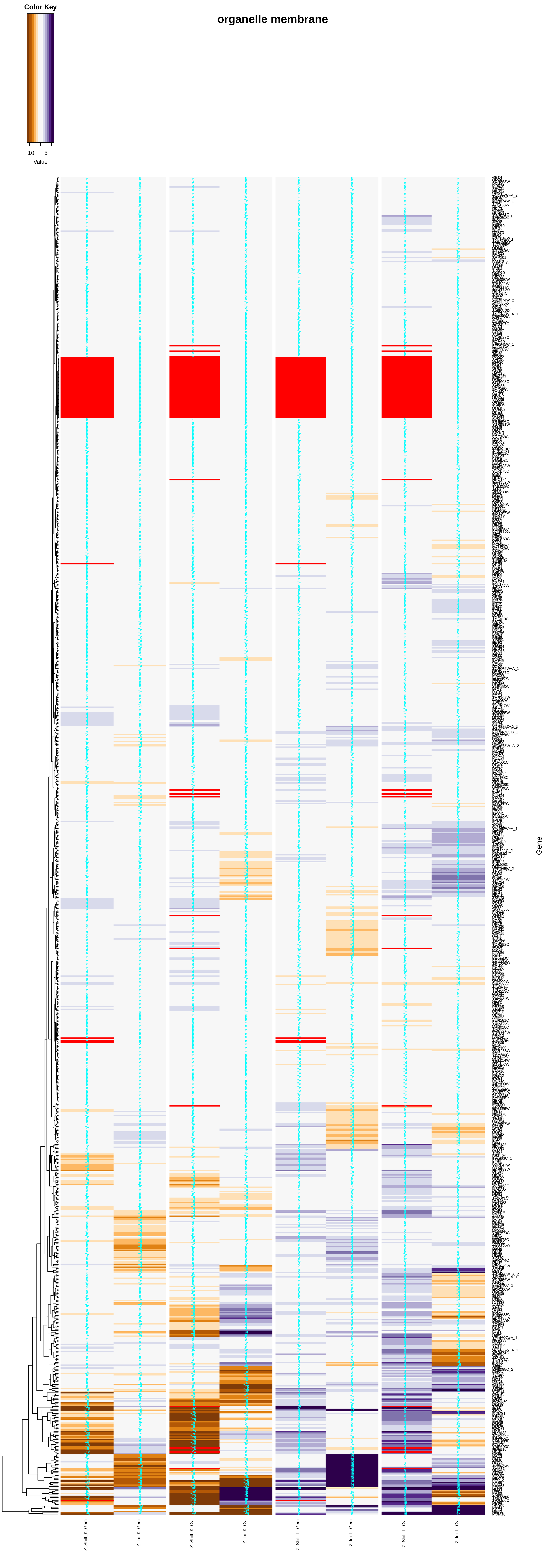
organelle membrane
Color Key
−10
5
Value
Gene
Z_Shift_K_Gem
Z_Im_K_Gem
Z_Shift_K_Cyt
Z_Im_K_Cyt
Z_Shift_L_Gem
Z_Im_L_Gem
Z_Shift_L_Cyt
Z_Im_L_Cyt

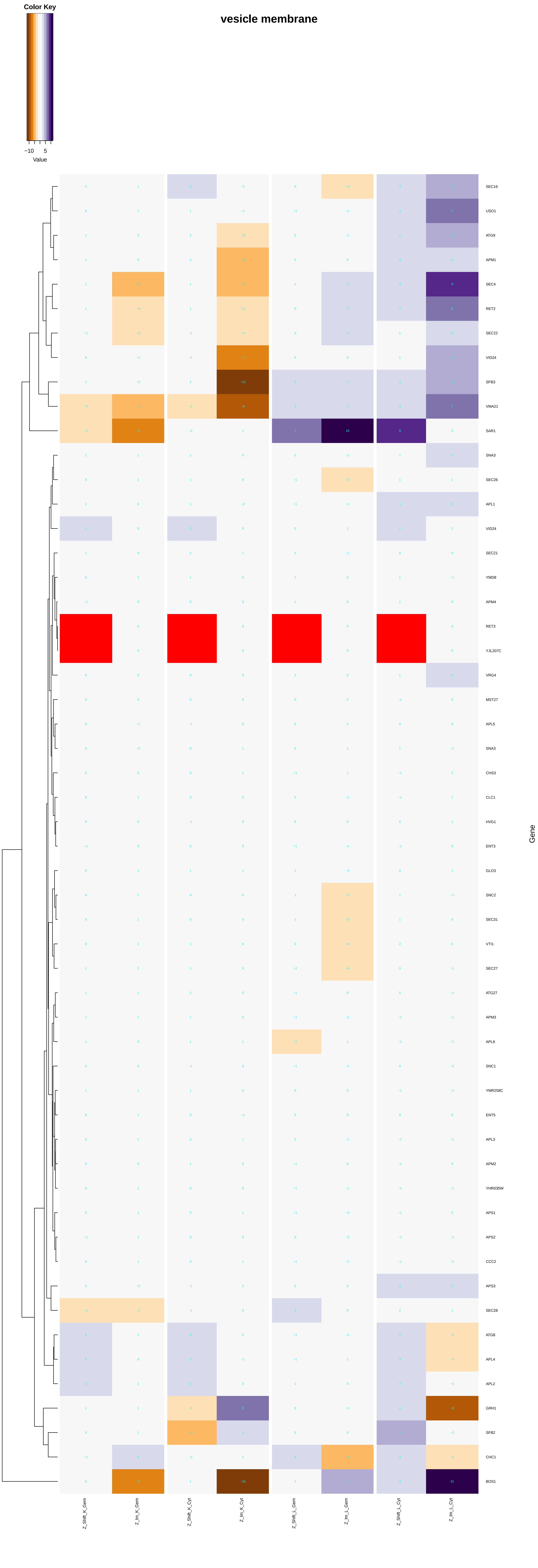
vesicle membrane
Color Key
−10
5
Value
SEC16
USO1
ATG9
APM1
SEC4
RET2
SEC22
VID24
SFB3
VMA21
SAR1
SNA4
SEC26
APL1
VID24
SEC21
YMD8
APM4
RET3
YJL207C
VRG4
MST27
APL5
SNA3
CHS3
CLC1
HVG1
ENT3
GLO3
SNC2
SEC31
VTI1
SEC27
ATG27
APM3
APL6
SNC1
YMR258C
ENT5
APL3
APM2
YHR035W
APS1
APS2
CCC2
APS3
SEC28
ATG8
APL4
APL2
GRH1
SFB2
CHC1
BOS1
Gene
Z_Shift_K_Gem
Z_Im_K_Gem
Z_Shift_K_Cyt
Z_Im_K_Cyt
Z_Shift_L_Gem
Z_Im_L_Gem
Z_Shift_L_Cyt
Z_Im_L_Cyt

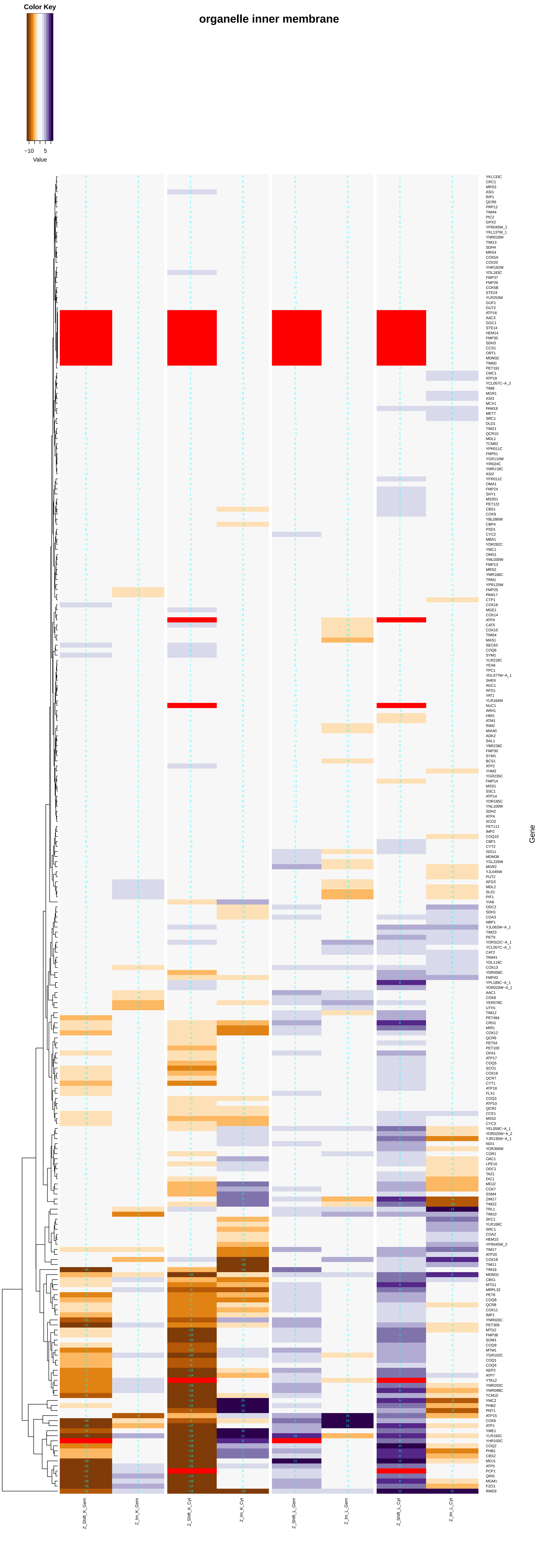
organelle inner membrane
Color Key
−10
5
Value
Gene
Z_Shift_K_Gem
Z_Im_K_Gem
Z_Shift_K_Cyt
Z_Im_K_Cyt
Z_Shift_L_Gem
Z_Im_L_Gem
Z_Shift_L_Cyt
Z_Im_L_Cyt

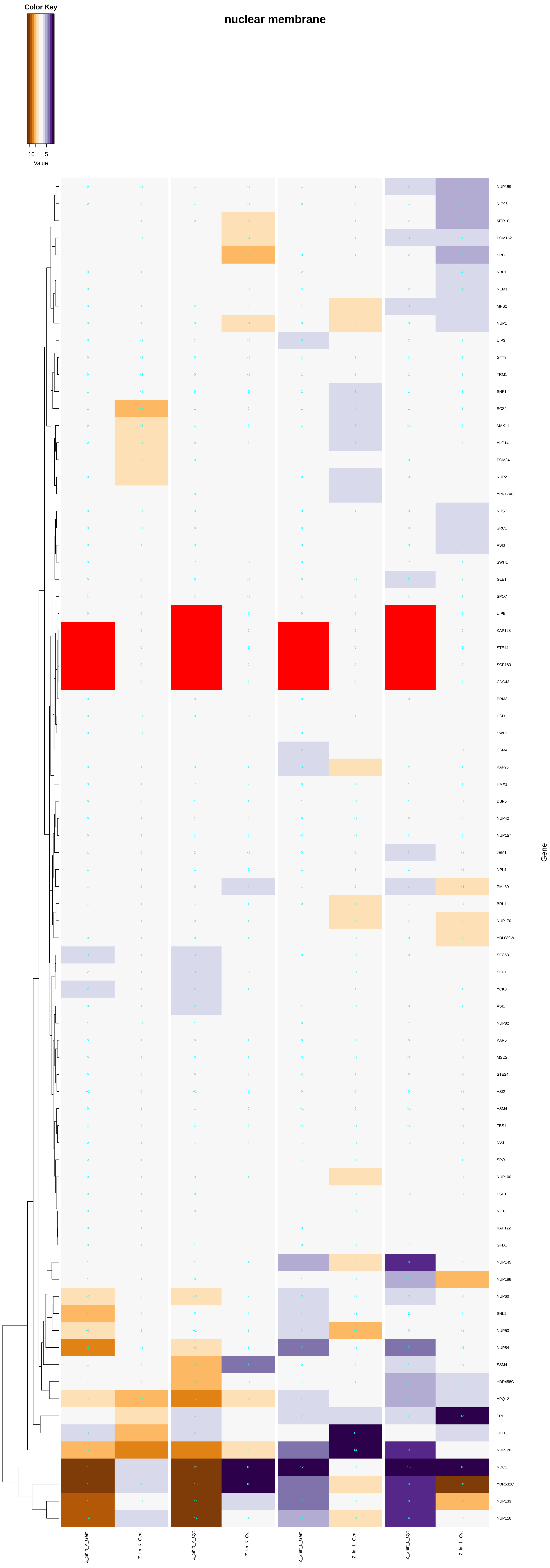
nuclear membrane
Color Key
−10
5
Value
Gene
NUP159
NIC96
MTR10
POM152
SRC1
NBP1
NEM1
MPS2
NUP1
UIP3
GTT3
TRM1
SNF1
SCS2
MAK11
ALG14
POM34
NUP2
YPR174C
NUS1
SRC1
ASI3
SWH1
GLE1
SPO7
UIP5
KAP123
STE14
SCP160
CDC42
PRM3
HSD1
SWH1
CSM4
KAP95
HMX1
DBP5
NUP42
NUP157
JEM1
NPL4
PML39
BRL1
NUP170
YDL089W
SEC63
SEH1
YCK3
ASI1
NUP82
KAR5
MSC2
STE24
ASI2
ASM4
TBS1
NVJ1
SPO1
NUP100
PSE1
NEJ1
KAP122
GFD1
NUP145
NUP188
NUP60
SNL1
NUP53
NUP84
SSM4
YDR458C
APQ12
TRL1
OPI1
NUP120
NDC1
YDR532C
NUP133
NUP116
Z_Shift_K_Gem
Z_Im_K_Gem
Z_Shift_K_Cyt
Z_Im_K_Cyt
Z_Shift_L_Gem
Z_Im_L_Gem
Z_Shift_L_Cyt
Z_Im_L_Cyt

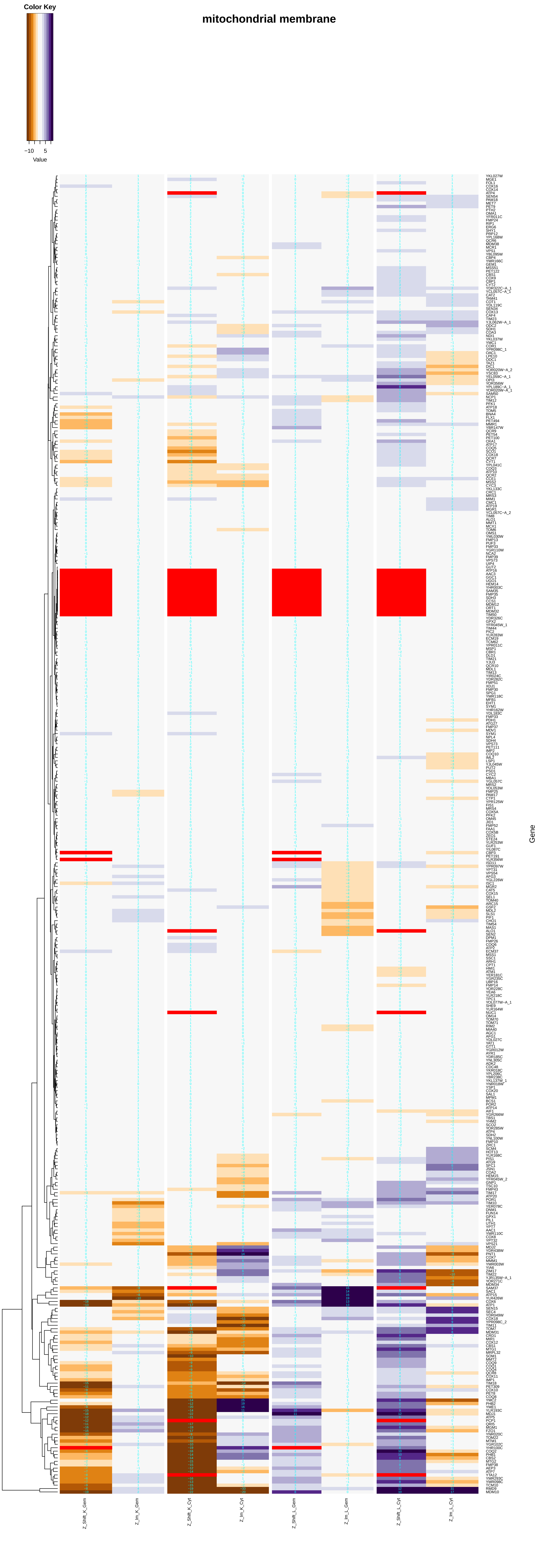
mitochondrial membrane
Color Key
−10
5
Value
Gene
Z_Shift_K_Gem
Z_Im_K_Gem
Z_Shift_K_Cyt
Z_Im_K_Cyt
Z_Shift_L_Gem
Z_Im_L_Gem
Z_Shift_L_Cyt
Z_Im_L_Cyt

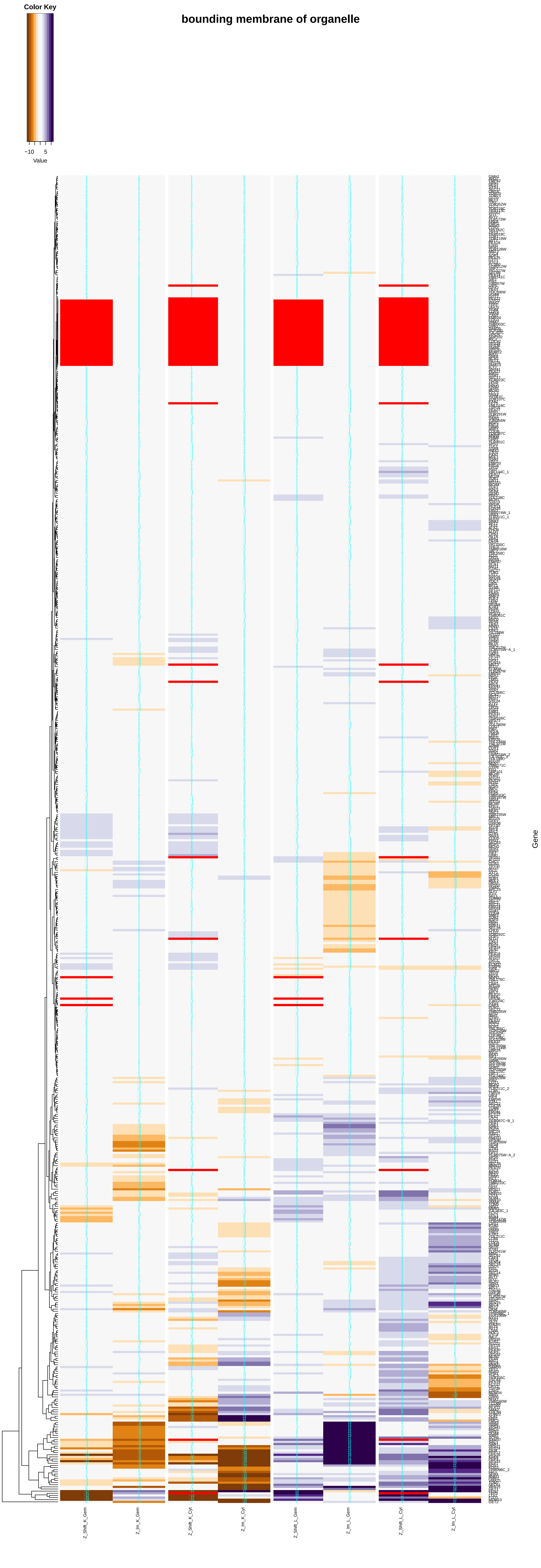
bounding membrane of organelle
Color Key
−10
5
Value
Gene
Z_Shift_K_Gem
Z_Im_K_Gem
Z_Shift_K_Cyt
Z_Im_K_Cyt
Z_Shift_L_Gem
Z_Im_L_Gem
Z_Shift_L_Cyt
Z_Im_L_Cyt

cytoplasmic vesicle membrane

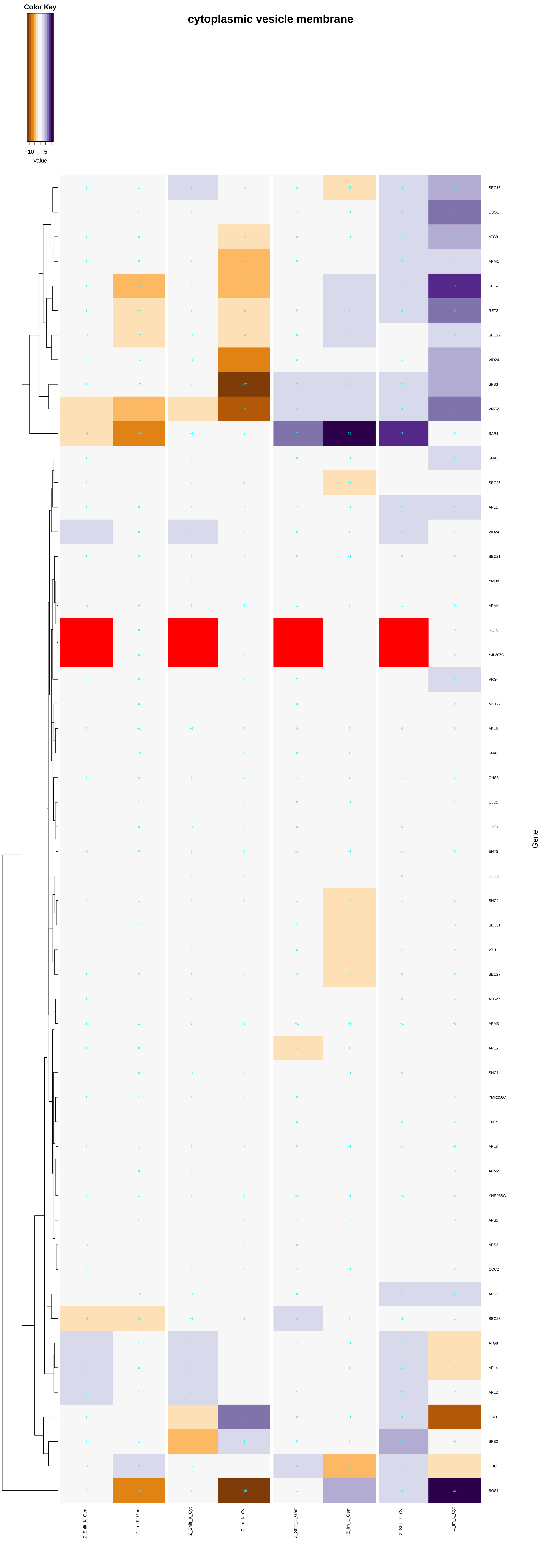

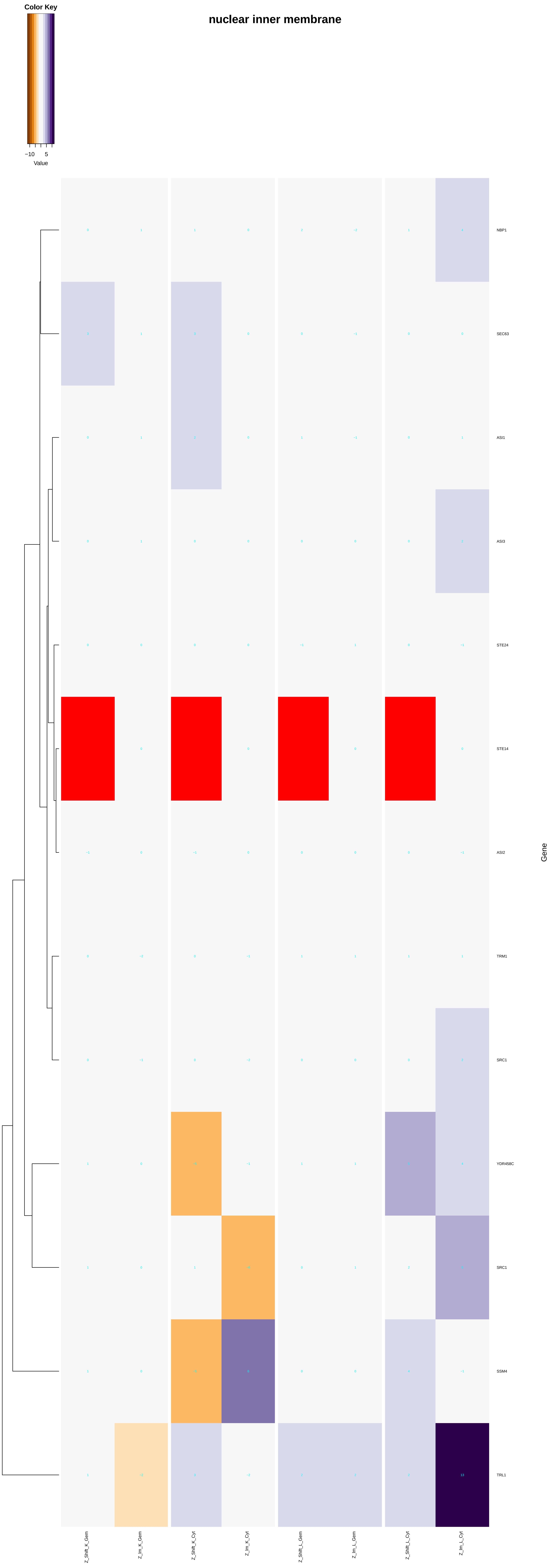
nuclear inner membrane
Color Key
−10
5
Value
NBP1
SEC63
ASI1
ASI3
STE24
STE14
ASI2
TRM1
SRC1
YDR458C
SRC1
SSM4
TRL1
Gene
Z_Shift_K_Gem
Z_lm_K_Gem
Z_Shift_K_Cyt
Z_lm_K_Cyt
Z_Shift_L_Gem
Z_lm_L_Gem
Z_Shift_L_Cyt
Z_lm_L_Cyt

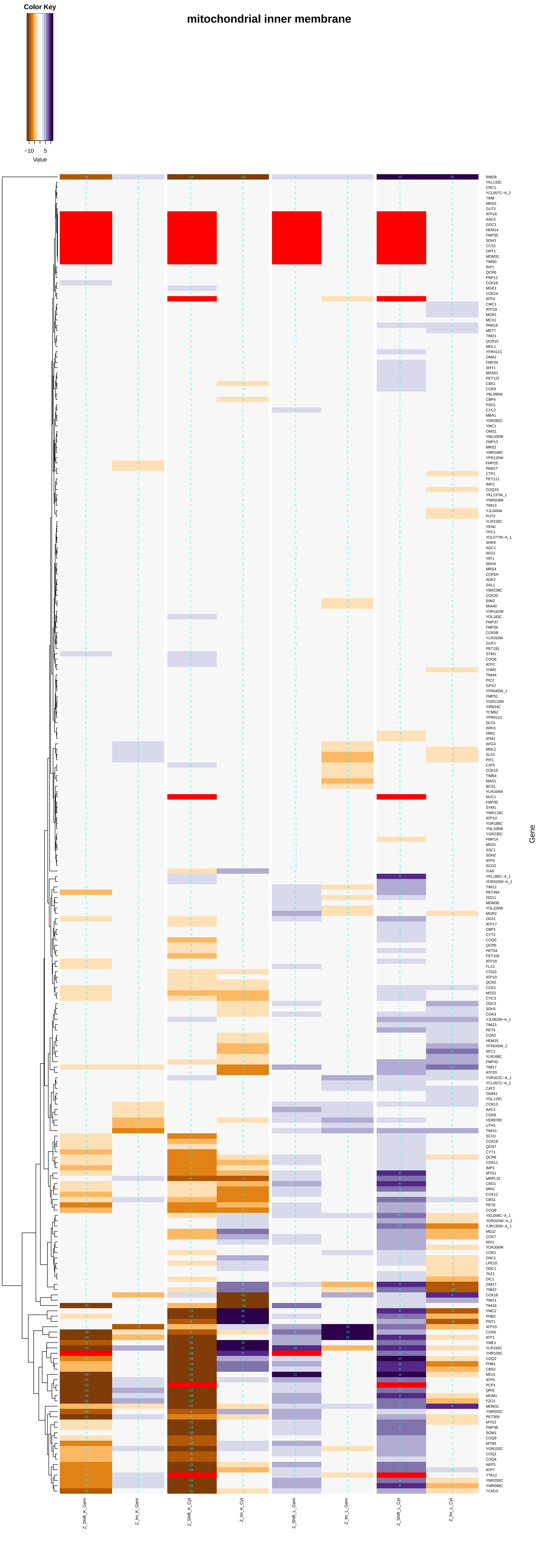

mitochondrial inner membrane
Color Key
−10
5
Value
Gene
Z_Shift_K_Gem
Z_Im_K_Gem
Z_Shift_K_Cyt
Z_Im_K_Cyt
Z_Shift_L_Gem
Z_Im_L_Gem
Z_Shift_L_Cyt
Z_Im_L_Cyt

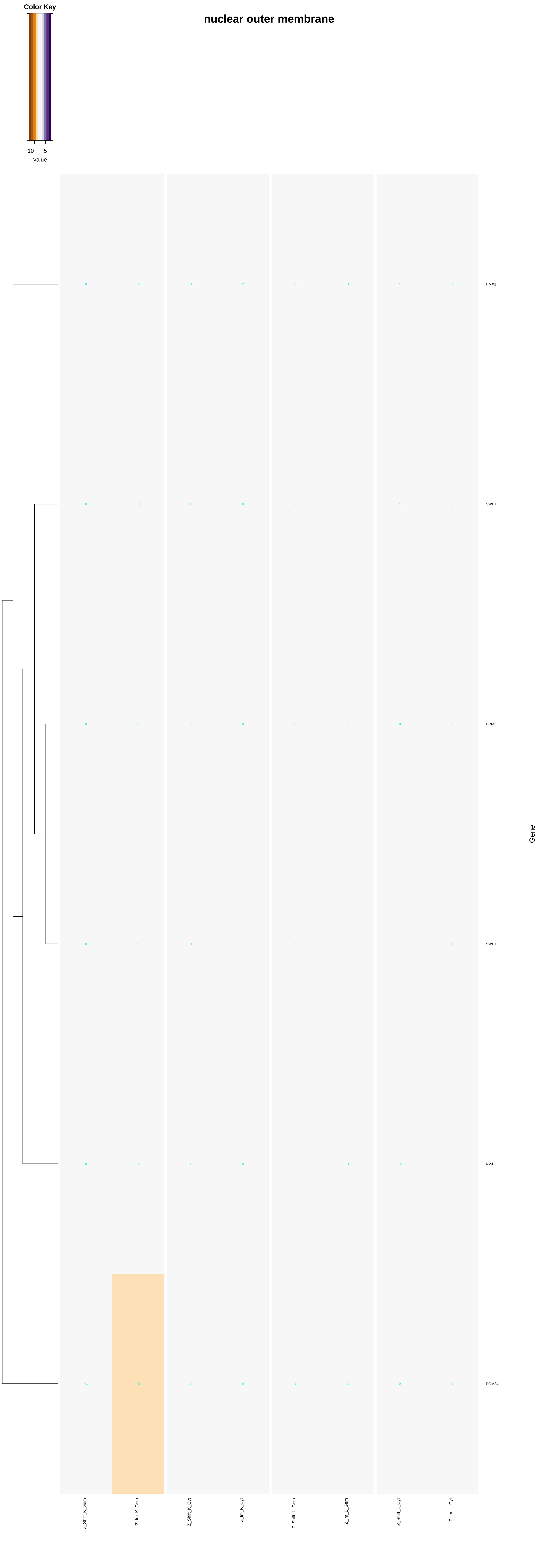

Color Key
−10
5
Value
nuclear outer membrane
HMX1
SWH1
PRM3
SWH1
NVJ1
POM34
Gene
Z_Shift_K_Gem
Z_Im_K_Gem
Z_Shift_K_Cyt
Z_Im_K_Cyt
Z_Shift_L_Gem
Z_Im_L_Gem
Z_Shift_L_Cyt
Z_Im_L_Cyt

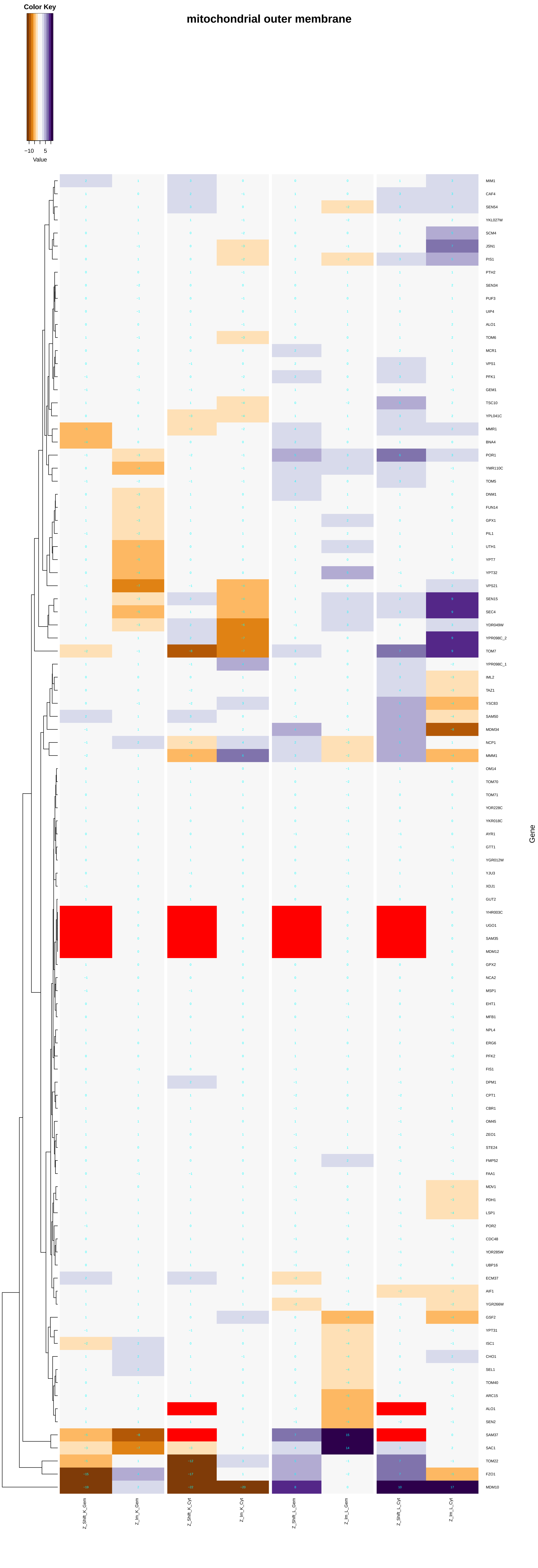
mitochondrial outer membrane
Color Key
-10
5
Value
MIM1
CAF4
SEN54
YKL027W
SCM4
JSN1
PIS1
PTH2
SEN34
PUF3
UIP4
ALO1
TOM6
MCR1
VPS1
PFK1
GEM1
TSC10
YPL041C
MMR1
BNA4
POR1
YMR110C
TOM5
DNM1
FUN14
GPX1
PIL1
UTH1
YPT7
YPT32
VPS21
SEN15
SEC4
YDR049W
YPR098C_2
TOM7
YPR098C_1
IML2
TAZ1
YSC83
SAM50
MDM34
NCP1
MMM1
OM14
TOM70
TOM71
YOR228C
YKR018C
AYR1
GTT1
YGR012W
YJU3
XDJ1
GUT2
YHR003C
UGO1
SAM35
MDM12
GPX2
NCA2
MSP1
EHT1
MFB1
NPL4
ERG6
PFK2
FIS1
DPM1
CPT1
CBR1
OM45
ZEO1
STE24
FMP52
FAA1
MDV1
PDH1
LSP1
POR2
CDC48
YOR285W
UBP16
ECM37
AIF1
YGR266W
GSF2
YPT31
ISC1
CHO1
SEL1
TOM40
ARC15
ALO1
SEN2
SAM37
SAC1
TOM22
FZO1
MDM10
Gene
Z_Shift_K_Gem
Z_Im_K_Gem
Z_Shift_K_Cyt
Z_Im_K_Cyt
Z_Shift_L_Gem
Z_Im_L_Gem
Z_Shift_L_Cyt
Z_Im_L_Cyt

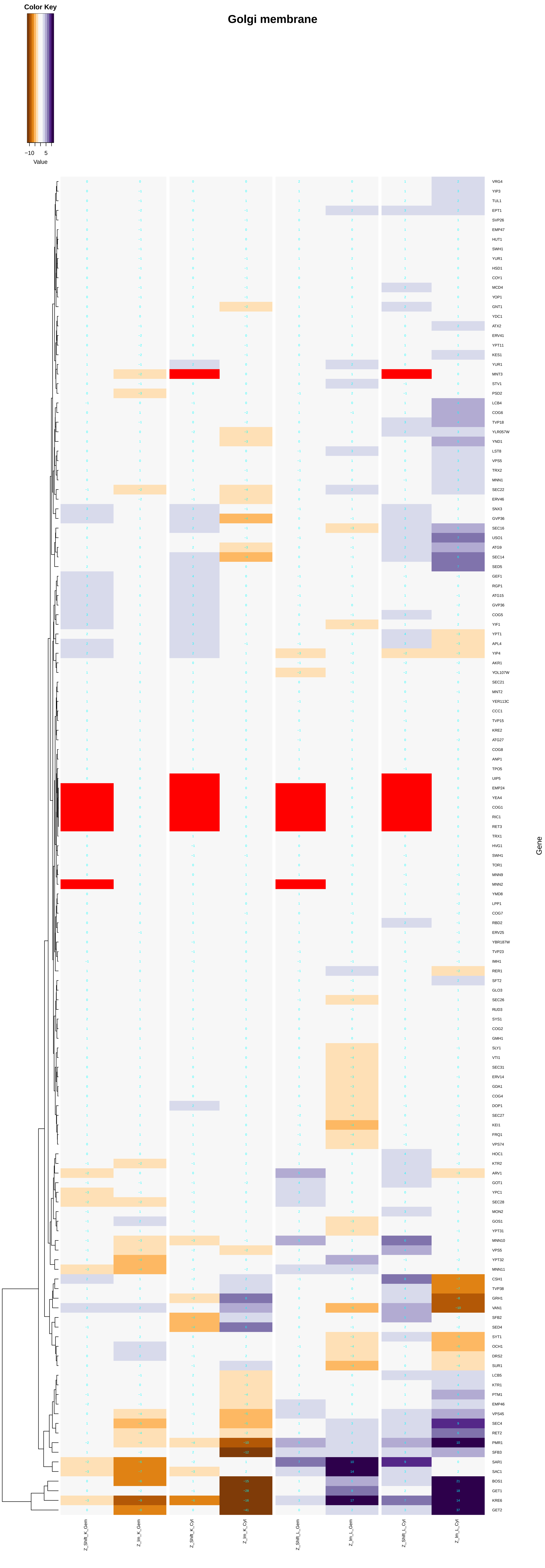
Color Key
−10 5
Value
Golgi membrane
VRG4
YIP3
TUL1
EPT1
SVP26
EMP47
HUT1
SWH1
YUR1
HSD1
COY1
MCD4
YOP1
GNT1
YDC1
ATX2
ERV41
YPT11
KES1
YUR1
MNT3
STV1
PSD2
LCB4
COG6
TVP18
YLR057W
YND1
LST8
VPS5
TRX2
MNN1
SEC22
ERV46
SNX3
GVP36
SEC16
USO1
ATG9
SEC14
SED5
GEF1
RGP1
ATG15
GVP36
COG5
YIF1
YPT1
APL4
YIP4
AKR1
YOL107W
SEC21
MNT2
YER113C
CCC1
TVP15
KRE2
ATG27
COG8
ANP1
TPO5
UIP5
EMP24
YEA4
COG1
RIC1
RET3
TRX1
HVG1
SWH1
TOR1
MNN9
MNN2
YMD8
LPP1
COG7
RBD2
ERV25
YBR187W
TVP23
IMH1
RER1
SFT2
GLO3
SEC26
RUD3
SYS1
COG2
GMH1
SLY1
VTI1
SEC31
ERV14
GDA1
COG4
DOP1
SEC27
KEI1
FRQ1
VPS74
HOC1
KTR2
ARV1
GOT1
YPC1
SEC28
MON2
GOS1
YPT31
MNN10
VPS5
YPT32
MNN11
CSH1
TVP38
GRH1
VAN1
SFB2
SED4
SYT1
OCH1
DRS2
SUR1
LCB5
KTR1
PTM1
EMP46
VPS45
SEC4
RET2
PMR1
SFB3
SAR1
SAC1
BOS1
GET1
KRE6
GET2
Gene
Z_Shift_K_Gem
Z_Im_K_Gem
Z_Shift_K_Cyt
Z_Im_K_Cyt
Z_Shift_L_Gem
Z_Im_L_Gem
Z_Shift_L_Cyt
Z_Im_L_Cyt

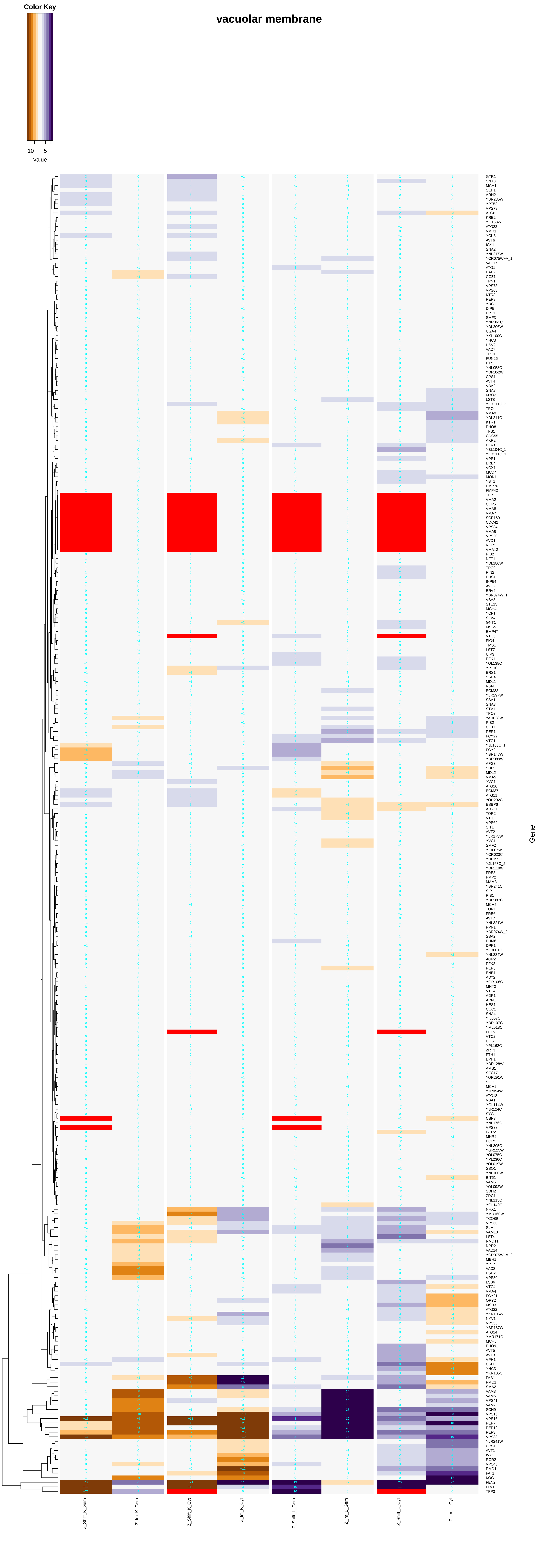
Color Key
Value
−10
5
vacuolar membrane
Gene
Z_Shift_K_Gem
Z_Im_K_Gem
Z_Shift_K_Cyt
Z_Im_K_Cyt
Z_Shift_L_Gem
Z_Im_L_Gem
Z_Shift_L_Cyt
Z_Im_L_Cyt

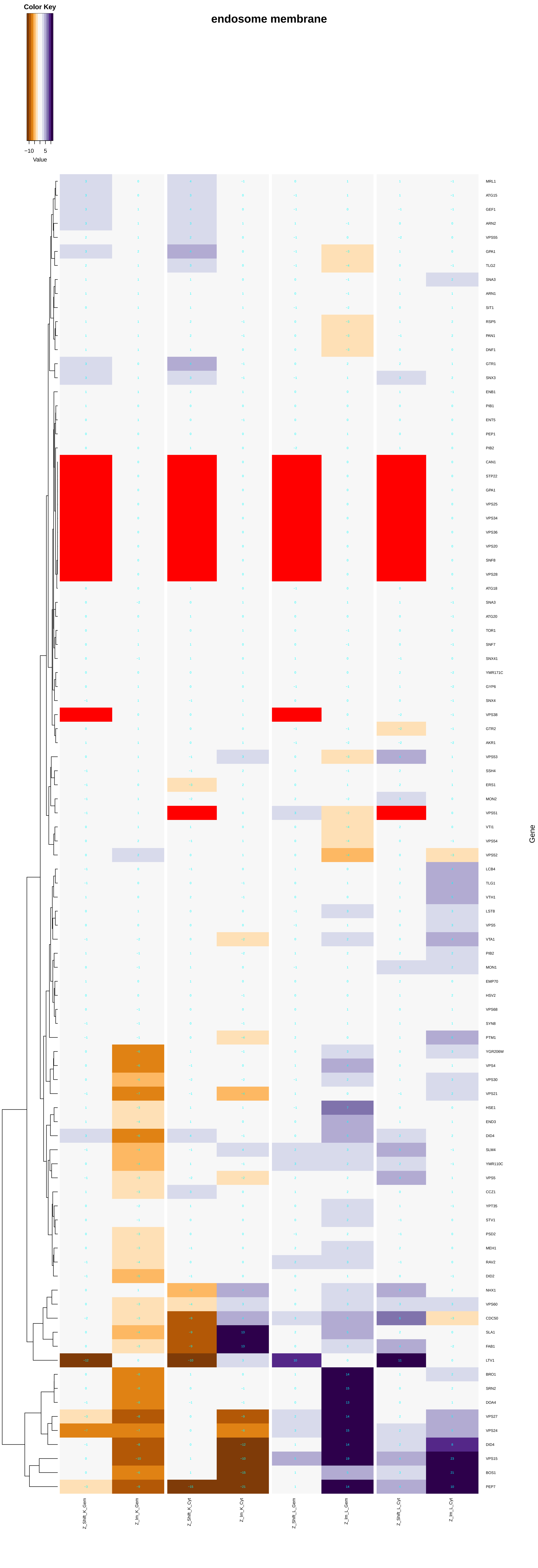
endosome membrane
Color Key
−10
5
Value
Gene
MRL1
ATG15
GEF1
ARN2
VPS55
GPA1
TLG2
SNA3
ARN1
SIT1
RSP5
PAN1
DNF1
GTR1
SNX3
ENB1
PIB1
ENT5
PEP1
PIB2
CAN1
STP22
GPA1
VPS25
VPS34
VPS36
VPS20
SNF8
VPS28
ATG18
SNA3
ATG20
TOR1
SNF7
SNX41
YMR171C
GYP6
SNX4
VPS38
GTR2
AKR1
VPS53
SSH4
ERS1
MON2
VPS51
VTI1
VPS54
VPS52
LCB4
TLG1
VTH1
LST8
VPS5
VTA1
PIB2
MON1
EMP70
HSV2
VPS68
SYN8
PTM1
YGR206W
VPS4
VPS30
VPS21
HSE1
END3
DID4
SLM4
YMR110C
VPS5
CCZ1
YPT35
STV1
PSD2
MEH1
RAV2
DID2
NHX1
VPS60
CDC50
SLA1
FAB1
LTV1
BRO1
SRN2
DOA4
VPS27
VPS24
DID4
VPS15
BOS1
PEP7
Z_Shift_K_Gem
Z_Im_K_Gem
Z_Shift_K_Cyt
Z_Im_K_Cyt
Z_Shift_L_Gem
Z_Im_L_Gem
Z_Shift_L_Cyt
Z_Im_L_Cyt

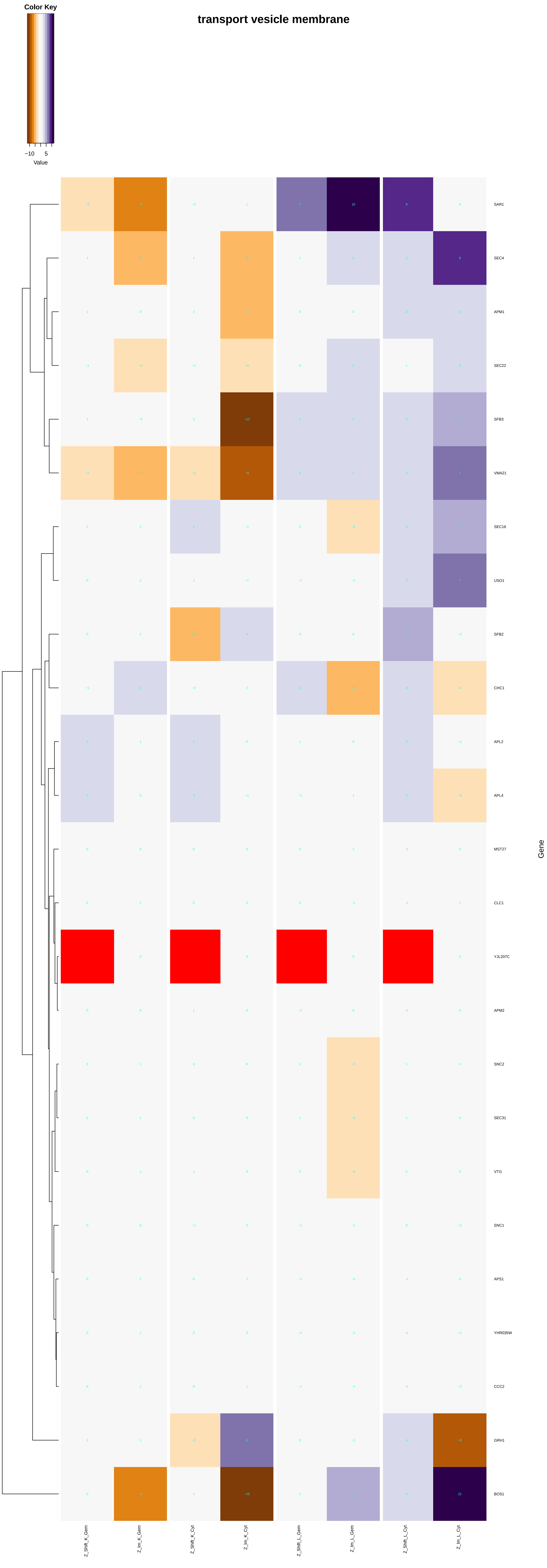
transport vesicle membrane
Color Key
−10
5
Value
SAR1
SEC4
APM1
SEC22
SFB3
VMA21
SEC16
USO1
SFB2
CHC1
APL2
APL4
MST27
CLC1
YJL207C
APM2
SNC2
SEC31
VTI1
SNC1
APS1
YHR035W
CCC2
GRH1
BOS1
Gene
Z_Shift_K_Gem
Z_Im_K_Gem
Z_Shift_K_Cyt
Z_Im_K_Cyt
Z_Shift_L_Gem
Z_Im_L_Gem
Z_Shift_L_Cyt
Z_Im_L_Cyt

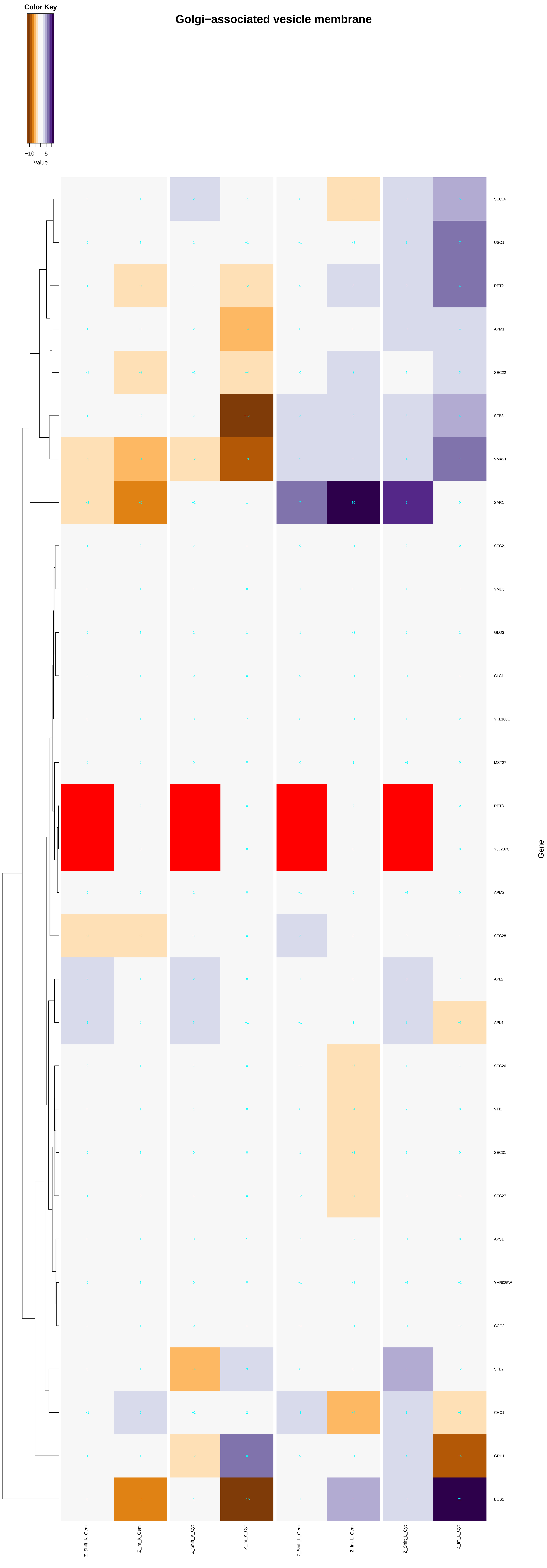
Golgi−associated vesicle membrane
Color Key
−10
5
Value
SEC16
USO1
RET2
APM1
SEC22
SFB3
VMA21
SAR1
SEC21
YMD8
GLO3
CLC1
YKL100C
MST27
RET3
YJL207C
APM2
SEC28
APL2
APL4
SEC26
VTI1
SEC31
SEC27
APS1
YHR035W
CCC2
SFB2
CHC1
GRH1
BOS1
Gene
Z_Shift_K_Gem
Z_Im_K_Gem
Z_Shift_K_Cyt
Z_Im_K_Cyt
Z_Shift_L_Gem
Z_Im_L_Gem
Z_Shift_L_Cyt
Z_Im_L_Cyt

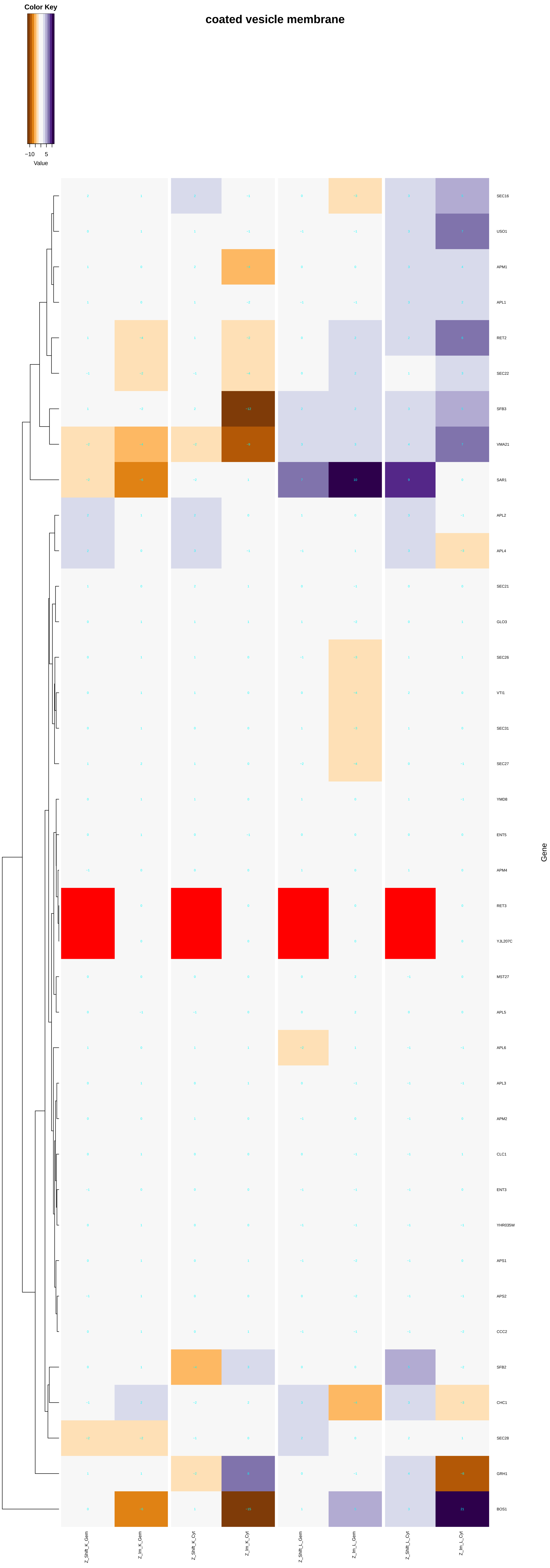
coated vesicle membrane
Color Key
−10
5
Value
SEC16
USO1
APM1
APL1
RET2
SEC22
SFB3
VMA21
SAR1
APL2
APL4
SEC21
GLO3
SEC26
VTI1
SEC31
SEC27
YMD8
ENT5
APM4
RET3
YJL207C
MST27
APL5
APL6
APL3
APM2
CLC1
ENT3
YHR035W
APS1
APS2
CCC2
SFB2
CHC1
SEC28
GRH1
BOS1
Gene
Z_Shift_K_Gem
Z_Im_K_Gem
Z_Shift_K_Cyt
Z_Im_K_Cyt
Z_Shift_L_Gem
Z_Im_L_Gem
Z_Shift_L_Cyt
Z_Im_L_Cyt

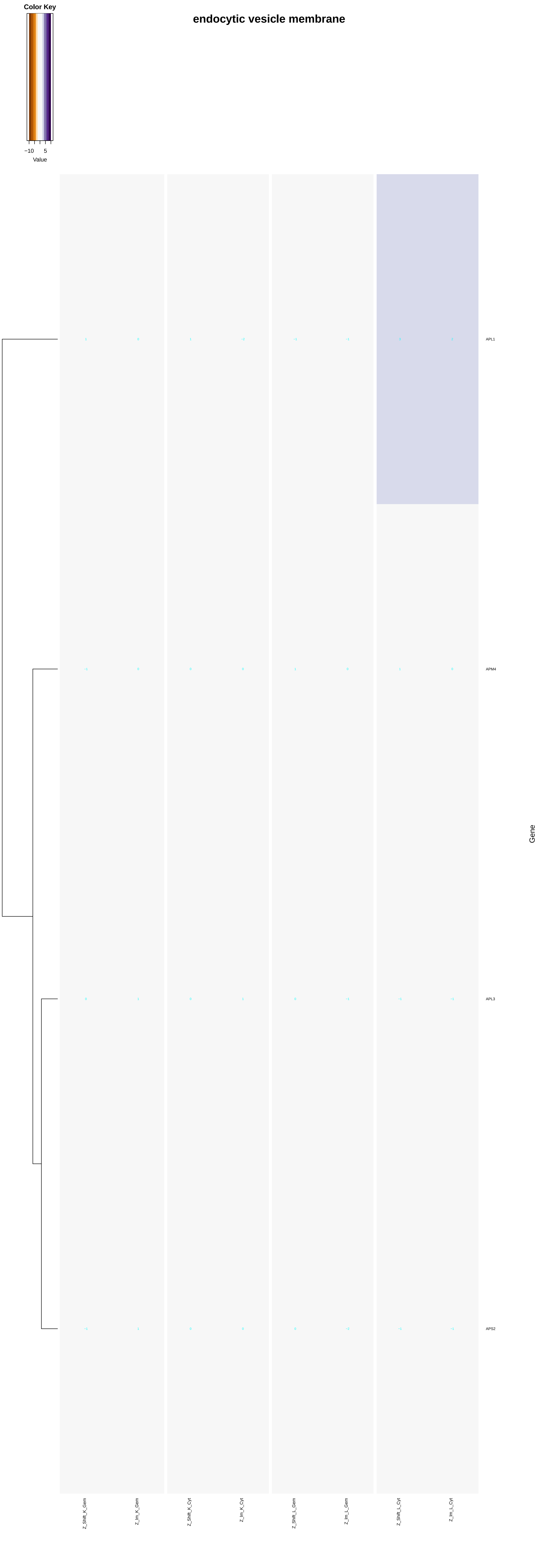
endocytic vesicle membrane
Color Key
−10
5
Value
APL1
APM4
APL3
APS2
Gene
Z_Shift_K_Gem
Z_Im_K_Gem
Z_Shift_K_Cyt
Z_Im_K_Cyt
Z_Shift_L_Gem
Z_Im_L_Gem
Z_Shift_L_Cyt
Z_Im_L_Cyt

rough endoplasmic reticulum membrane

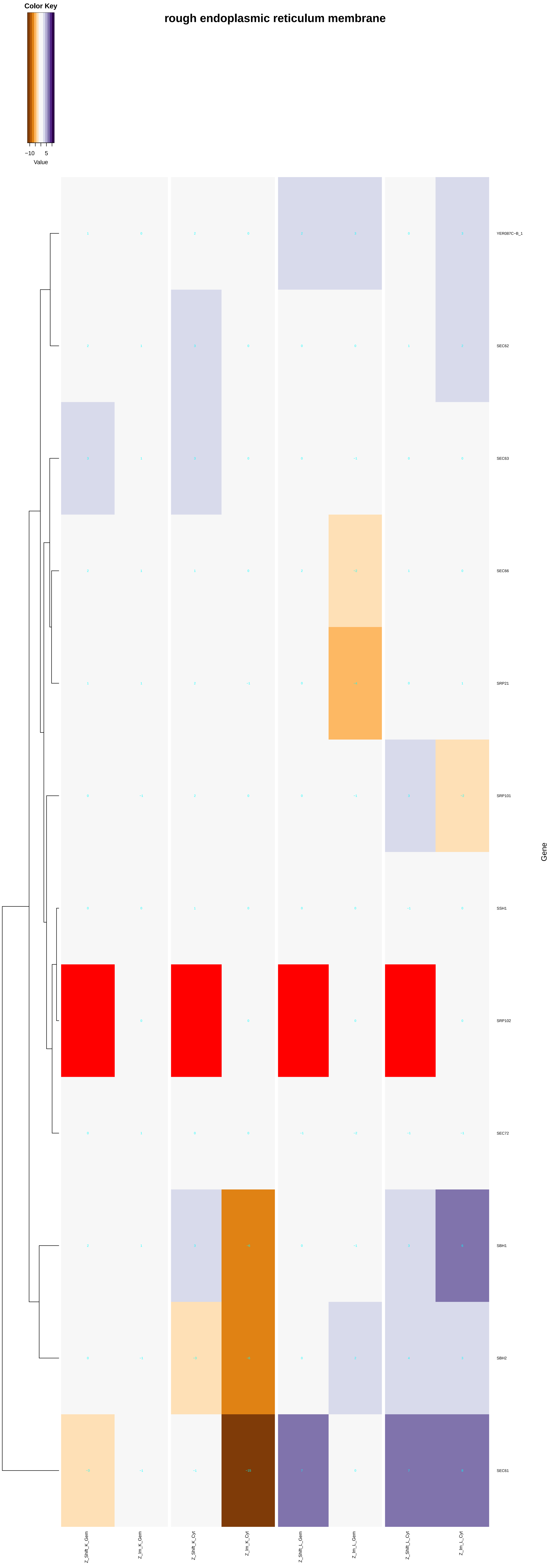

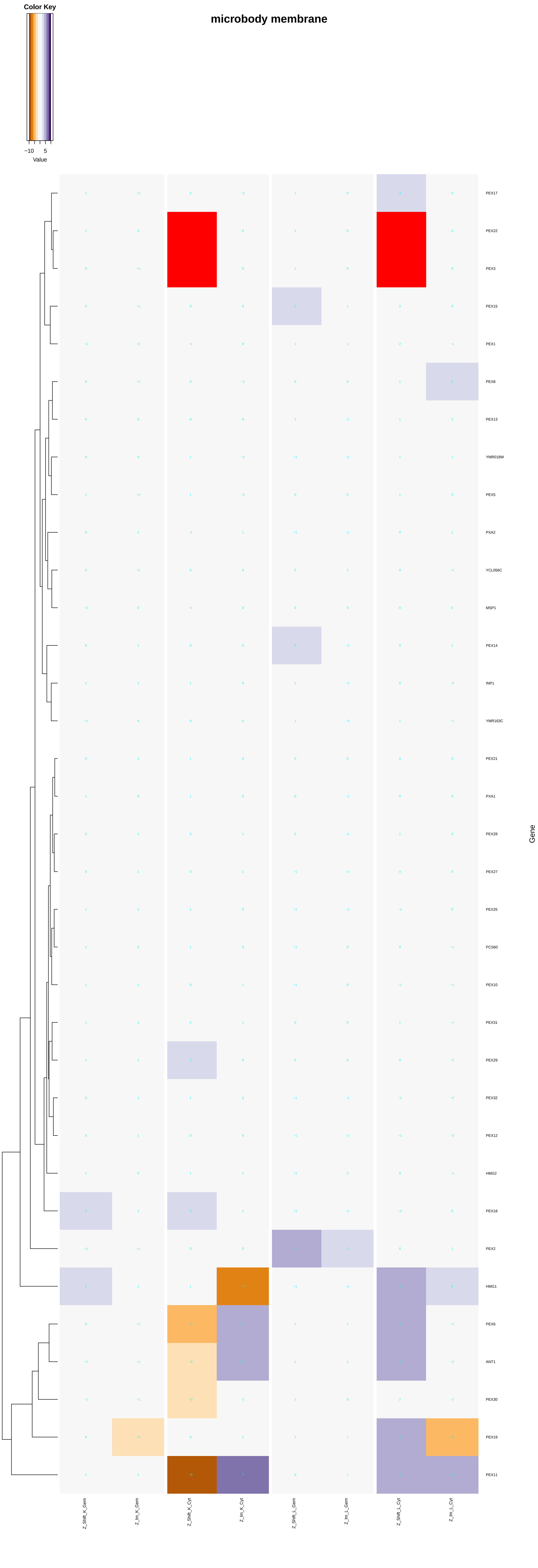
Color Key
−10
5
Value
microbody membrane
PEX17
PEX22
PEX3
PEX15
PEX1
PEX8
PEX13
YMR018W
PEX5
PXA2
YCL056C
MSP1
PEX14
INP1
YMR163C
PEX21
PXA1
PEX28
PEX27
PEX25
PCS60
PEX10
PEX31
PEX29
PEX32
PEX12
HMG2
PEX18
PEX2
HMG1
PEX6
ANT1
PEX30
PEX19
PEX11
Gene
Z_Shift_K_Gem
Z_Im_K_Gem
Z_Shift_K_Cyt
Z_Im_K_Cyt
Z_Shift_L_Gem
Z_Im_L_Gem
Z_Shift_L_Cyt
Z_Im_L_Cyt

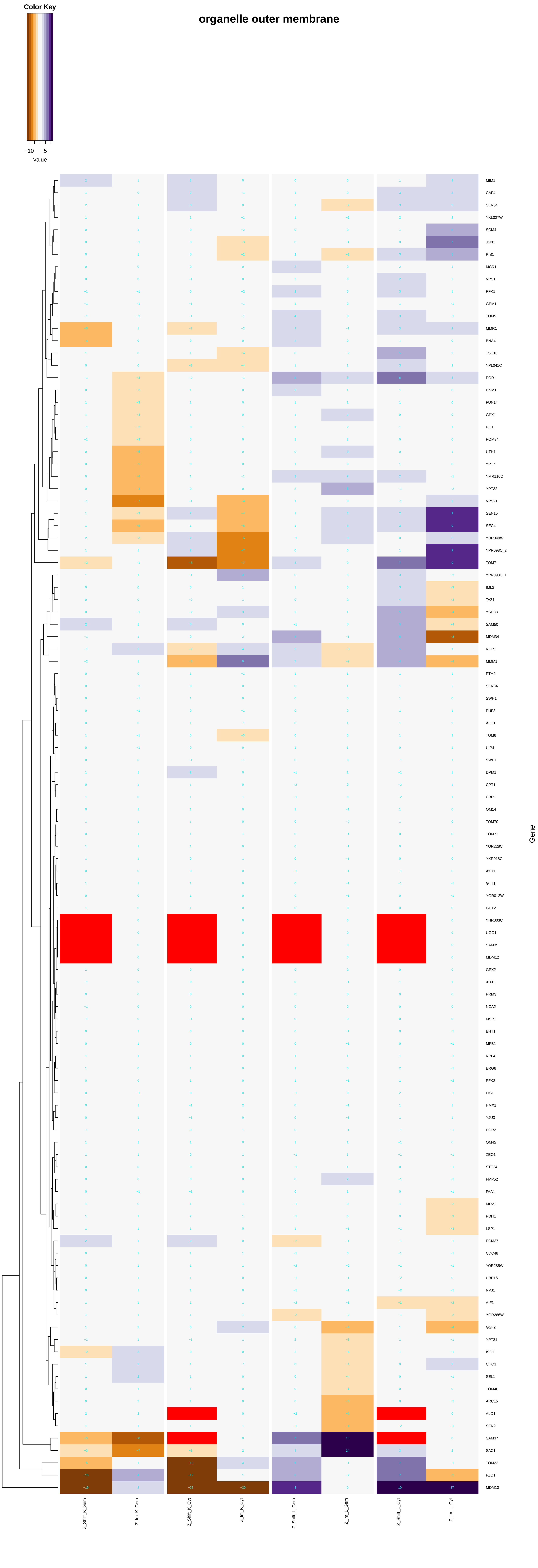

organelle outer membrane
Color Key
−10
5
Value
Gene
MIM1
CAF4
SEN54
YKL027W
SCM4
JSN1
PIS1
MCR1
VPS1
PFK1
GEM1
TOM5
MMR1
BNA4
TSC10
YPL041C
POR1
DNM1
FUN14
GPX1
PIL1
POM34
UTH1
YPT7
YMR110C
YPT32
VPS21
SEN15
SEC4
YDR049W
YPR098C_2
TOM7
YPR098C_1
IML2
TAZ1
YSC83
SAM50
MDM34
NCP1
MMM1
PTH2
SEN34
SWH1
PUF3
ALO1
TOM6
UIP4
SWH1
DPM1
CPT1
CBR1
OM14
TOM70
TOM71
YOR228C
YKR018C
AYR1
GTT1
YGR012W
GUT2
YHR003C
UGO1
SAM35
MDM12
GPX2
XDJ1
PRM3
NCA2
MSP1
EHT1
MFB1
NPL4
ERG6
PFK2
FIS1
HMX1
YJU3
POR2
OM45
ZEO1
STE24
FMP52
FAA1
MDV1
PDH1
LSP1
ECM37
CDC48
YOR285W
UBP16
NVJ1
AIF1
YGR266W
GSF2
YPT31
ISC1
CHO1
SEL1
TOM40
ARC15
ALO1
SEN2
SAM37
SAC1
TOM22
FZO1
MDM10
Z_Shift_K_Gem
Z_Im_K_Gem
Z_Shift_K_Cyt
Z_Im_K_Cyt
Z_Shift_L_Gem
Z_Im_L_Gem
Z_Shift_L_Cyt
Z_Im_L_Cyt

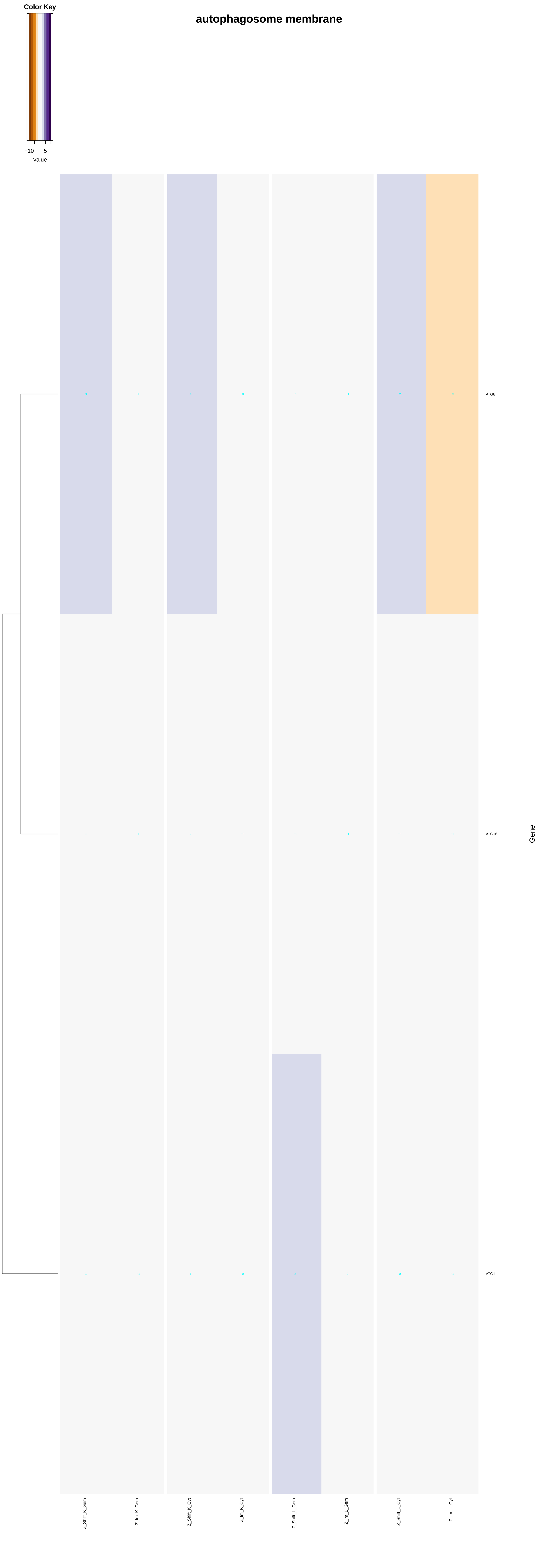
autophagosome membrane
Color Key
−10
5
Value
ATG8
ATG16
ATG1
Gene
Z_Shift_K_Gem
Z_Im_K_Gem
Z_Shift_K_Cyt
Z_Im_K_Cyt
Z_Shift_L_Gem
Z_Im_L_Gem
Z_Shift_L_Cyt
Z_Im_L_Cyt

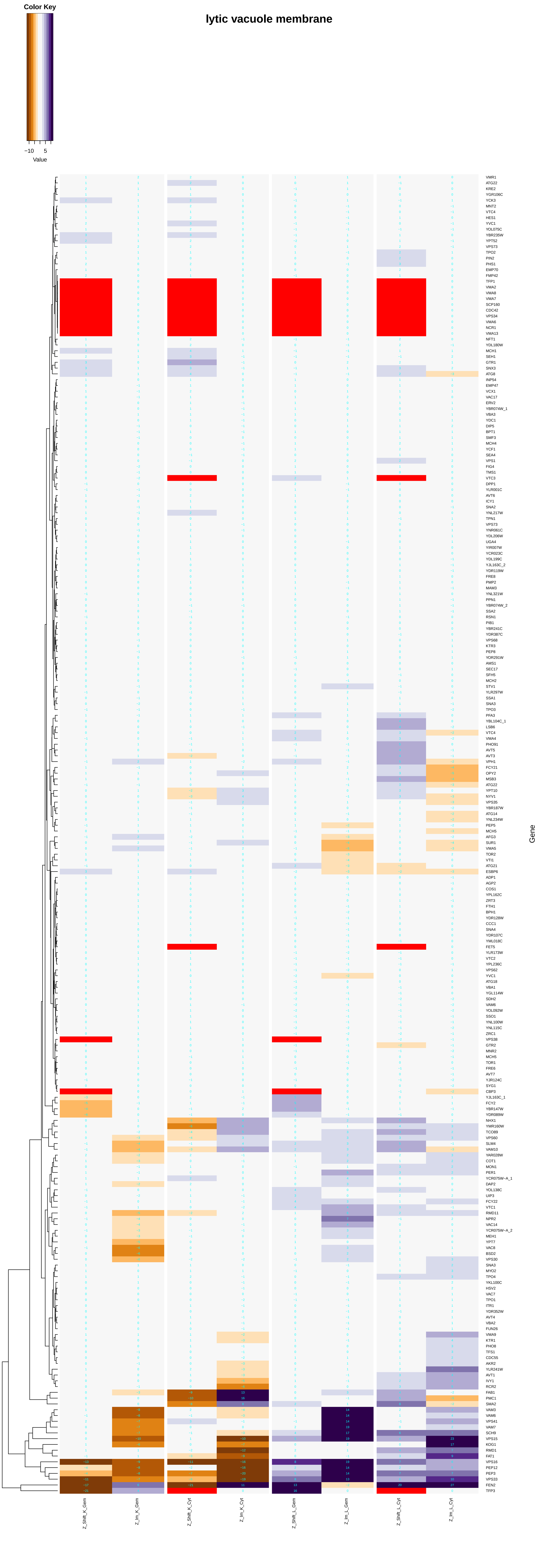

Color Key
−10
5
Value
lytic vacuole membrane
Gene
Z_Shift_K_Gem
Z_Im_K_Gem
Z_Shift_K_Cyt
Z_Im_K_Cyt
Z_Shift_L_Gem
Z_Im_L_Gem
Z_Shift_L_Cyt
Z_Im_L_Cyt

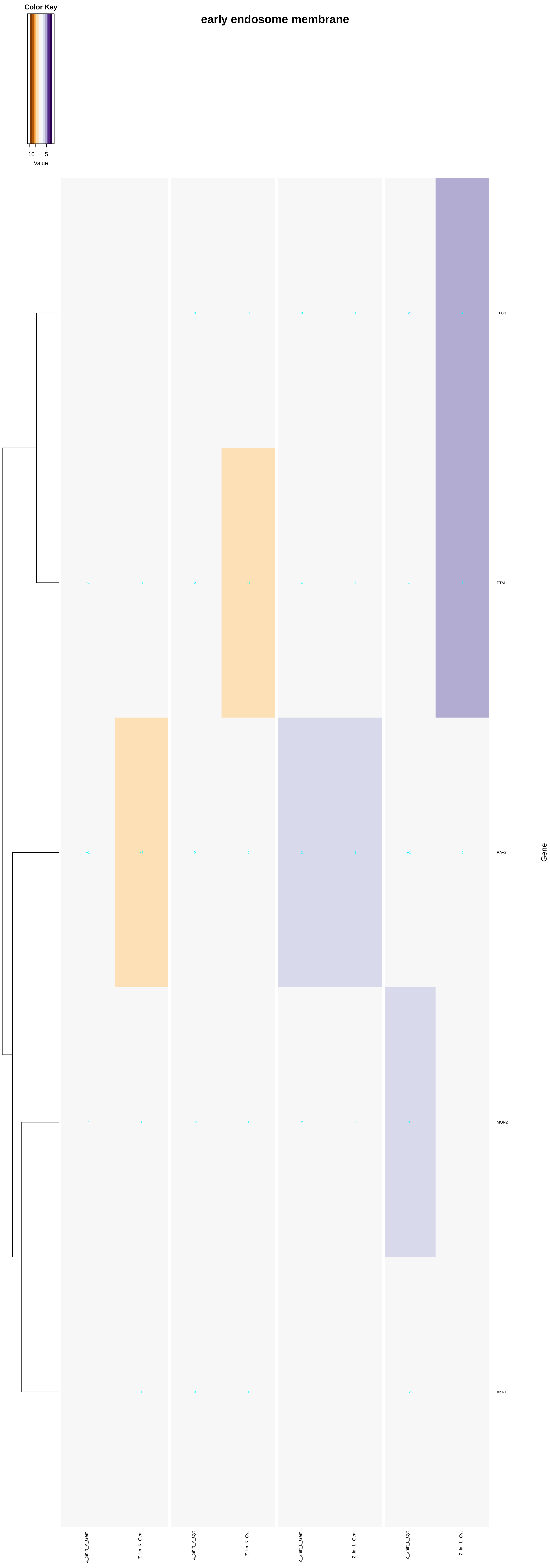
early endosome membrane
Color Key
−10
5
Value
TLG1
PTM1
RAV2
MON2
AKR1
Gene
Z_Shift_K_Gem
Z_Im_K_Gem
Z_Shift_K_Cyt
Z_Im_K_Cyt
Z_Shift_L_Gem
Z_Im_L_Gem
Z_Shift_L_Cyt
Z_Im_L_Cyt

late endosome membrane

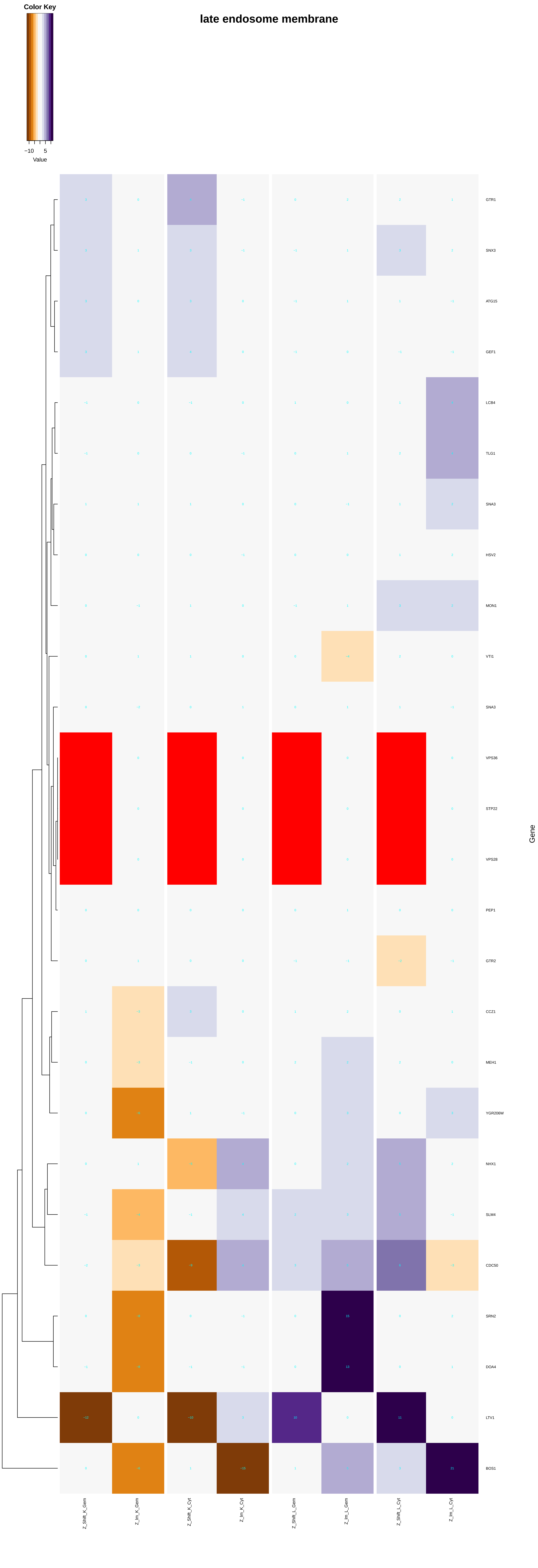

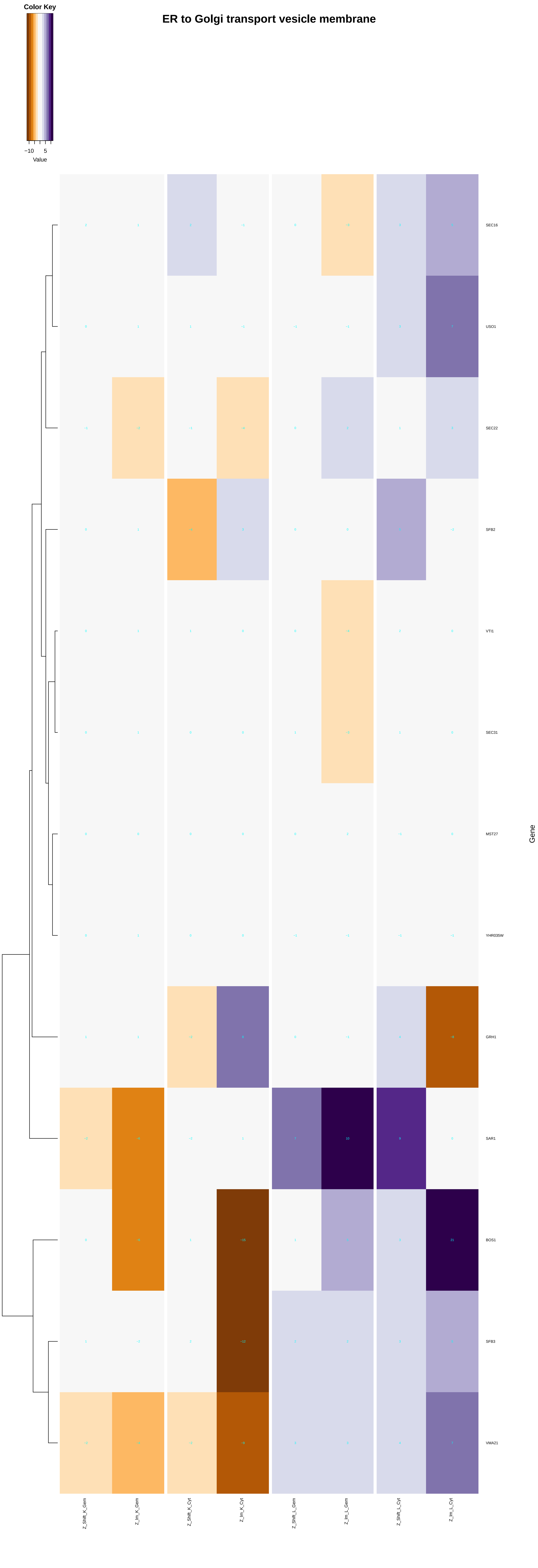
ER to Golgi transport vesicle membrane
Color Key
−10
5
Value
SEC16
USO1
SEC22
SFB2
VTI1
SEC31
MST27
YHR035W
GRH1
SAR1
BOS1
SFB3
VMA21
Gene
Z_Shift_K_Gem
Z_Im_K_Gem
Z_Shift_K_Cyt
Z_Im_K_Cyt
Z_Shift_L_Gem
Z_Im_L_Gem
Z_Shift_L_Cyt
Z_Im_L_Cyt

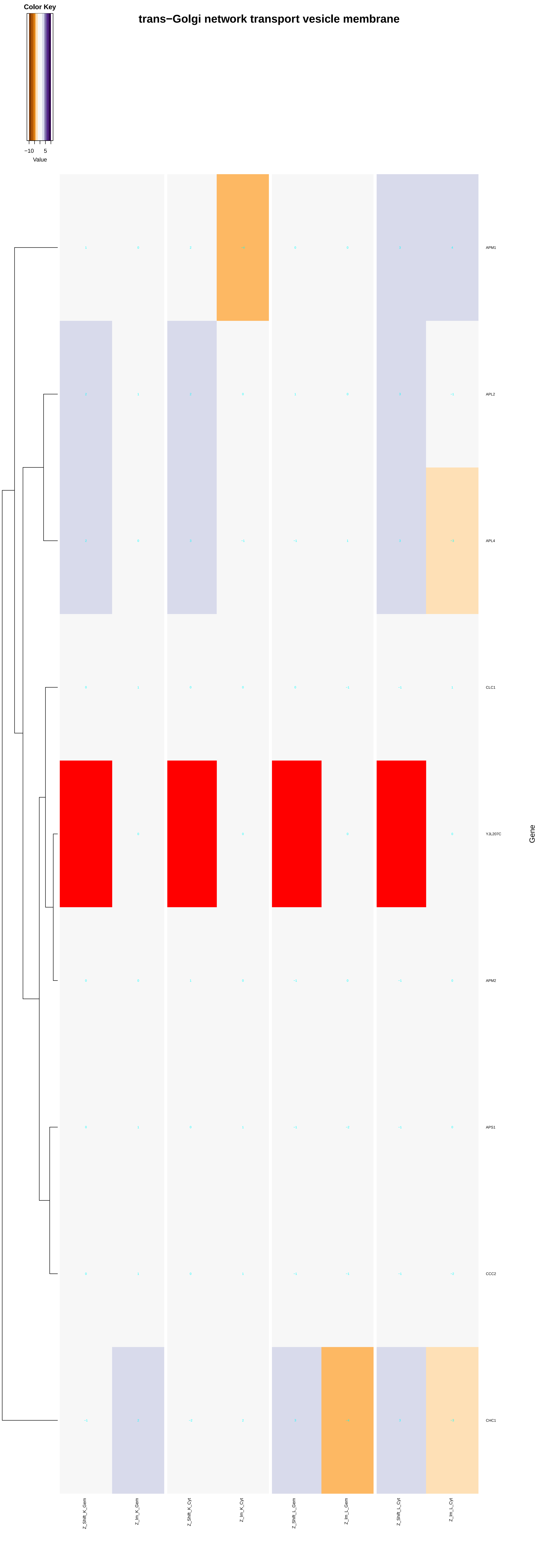
Color Key
−10
5
Value
trans−Golgi network transport vesicle membrane
APM1
APL2
APL4
CLC1
YJL207C
APM2
APS1
CCC2
CHC1
Gene
Z_Shift_K_Gem
Z_lm_K_Gem
Z_Shift_K_Cyt
Z_lm_K_Cyt
Z_Shift_L_Gem
Z_lm_L_Gem
Z_Shift_L_Cyt
Z_lm_L_Cyt

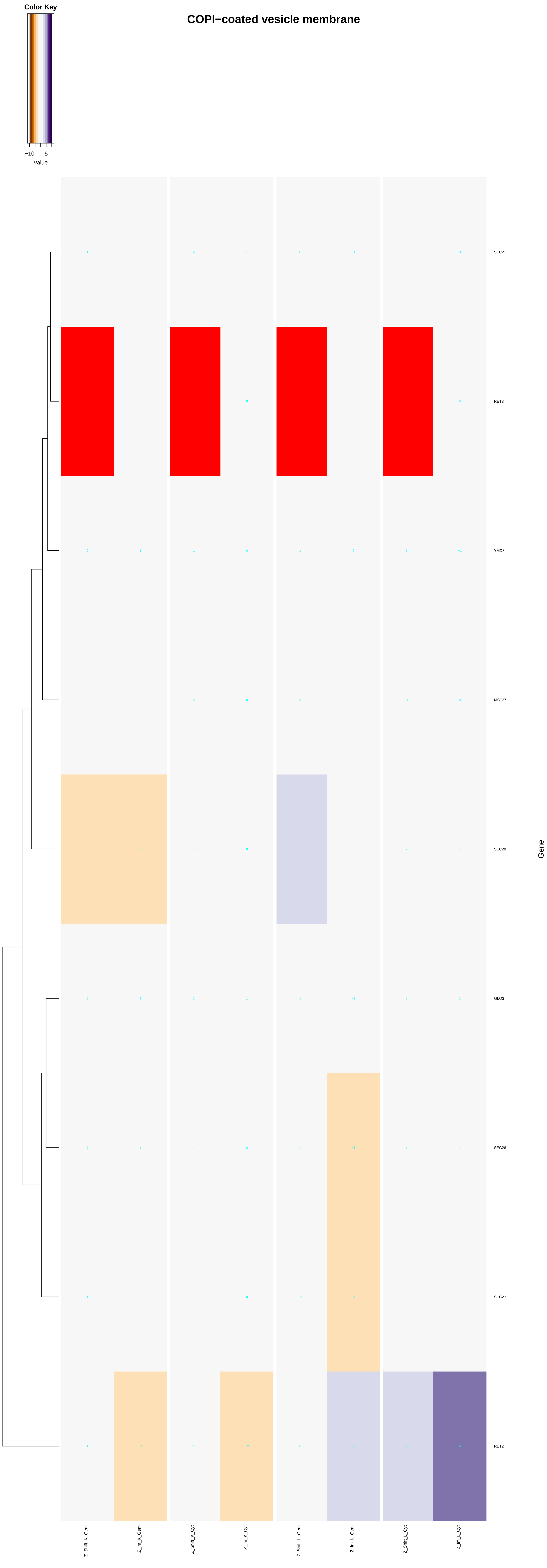

Color Key
−10
5
Value
COPI−coated vesicle membrane
SEC21
RET3
YMD8
MST27
SEC28
GLO3
SEC26
SEC27
RET2
Gene
Z_Shift_K_Gem
Z_Im_K_Gem
Z_Shift_K_Cyt
Z_Im_K_Cyt
Z_Shift_L_Gem
Z_Im_L_Gem
Z_Shift_L_Cyt
Z_Im_L_Cyt

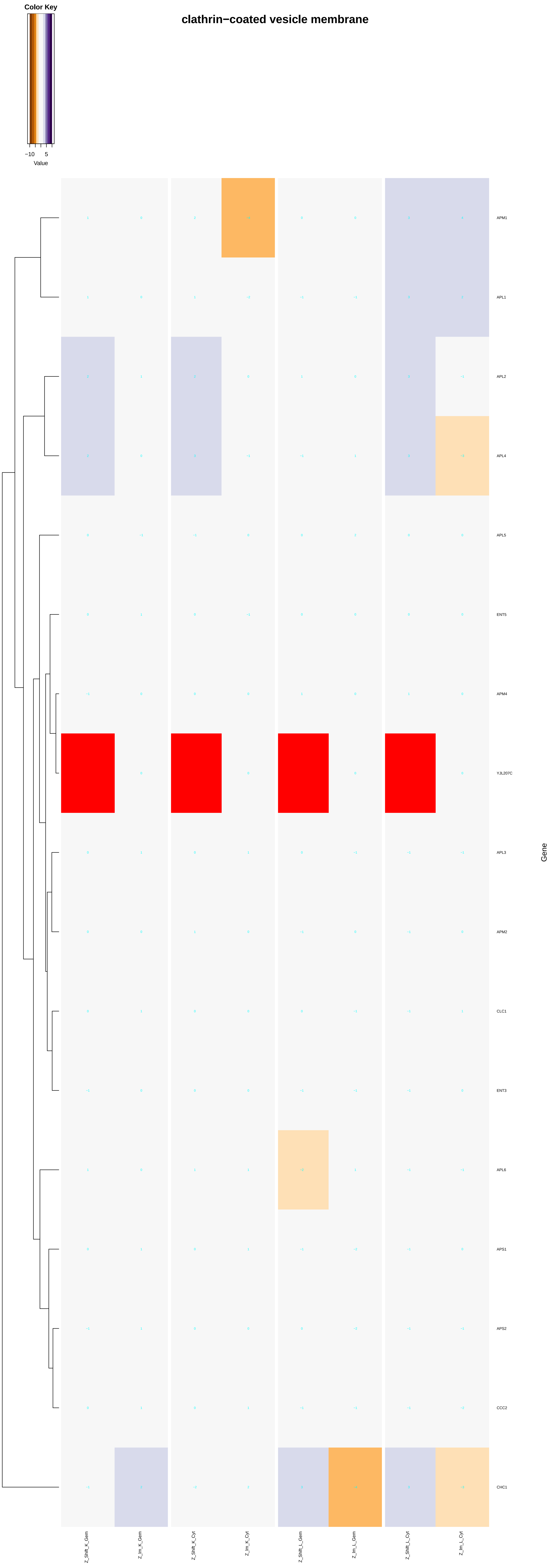
clathrin−coated vesicle membrane
Color Key
−10
5
Value
APM1
APL1
APL2
APL4
APL5
ENT5
APM4
YJL207C
APL3
APM2
CLC1
ENT3
APL6
APS1
APS2
CCC2
CHC1
Gene
Z_Shift_K_Gem
Z_Im_K_Gem
Z_Shift_K_Cyt
Z_Im_K_Cyt
Z_Shift_L_Gem
Z_Im_L_Gem
Z_Shift_L_Cyt
Z_Im_L_Cyt

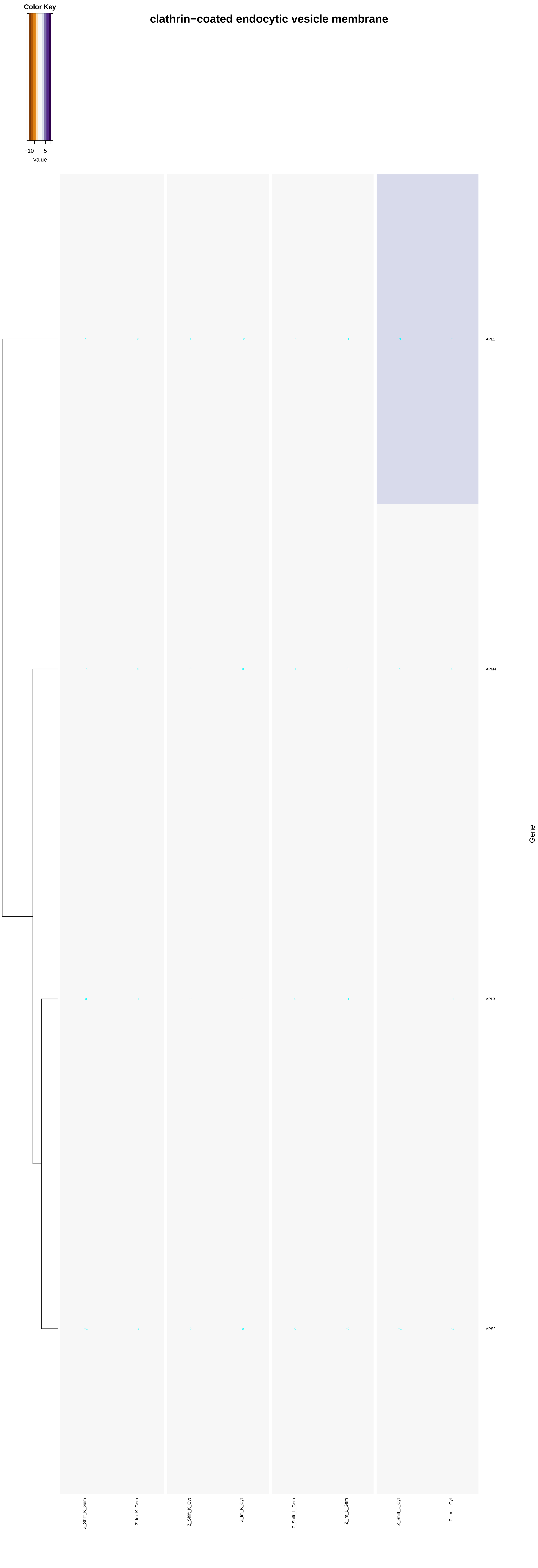
Color Key
−10
5
Value
clathrin−coated endocytic vesicle membrane
APL1
APM4
APL3
APS2
Gene
Z_Shift_K_Gem
Z_Im_K_Gem
Z_Shift_K_Cyt
Z_Im_K_Cyt
Z_Shift_L_Gem
Z_Im_L_Gem
Z_Shift_L_Cyt
Z_Im_L_Cyt

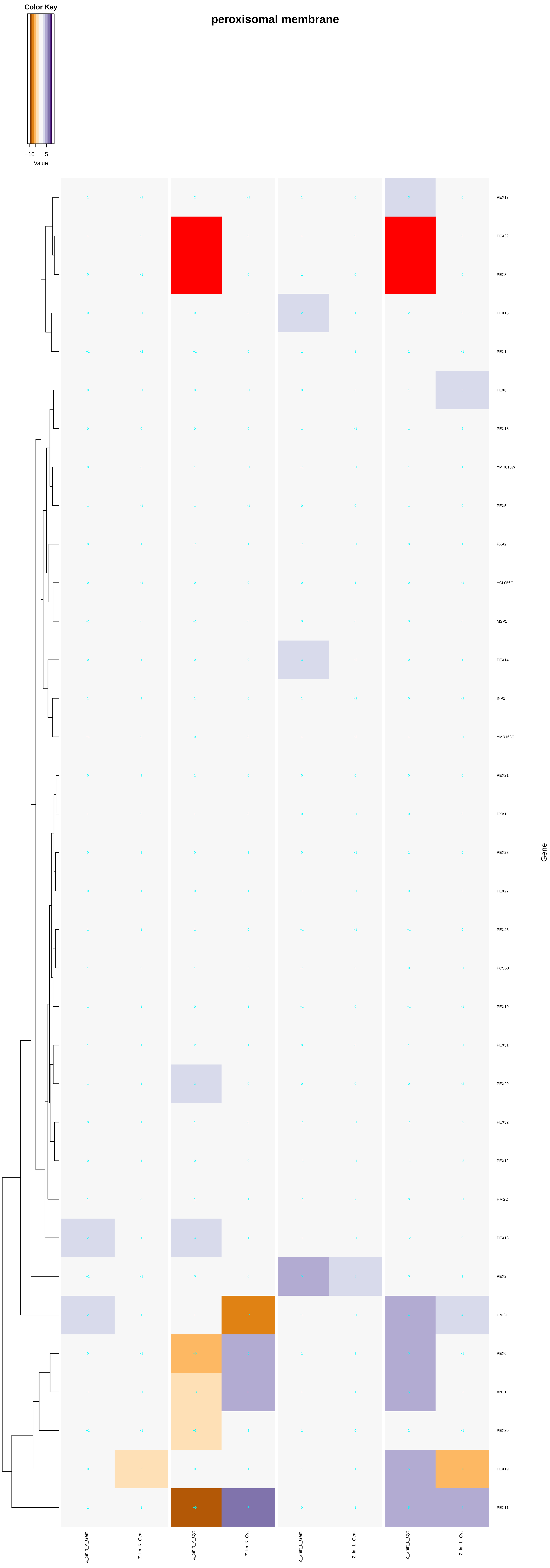

peroxisomal membrane
Color Key
−10
5
Value
Gene
PEX17
PEX22
PEX3
PEX15
PEX1
PEX8
PEX13
YMR018W
PEX5
PXA2
YCL056C
MSP1
PEX14
INP1
YMR163C
PEX21
PXA1
PEX28
PEX27
PEX25
PCS60
PEX10
PEX31
PEX29
PEX32
PEX12
HMG2
PEX18
PEX2
HMG1
PEX6
ANT1
PEX30
PEX19
PEX11
Z_Shift_K_Gem
Z_Im_K_Gem
Z_Shift_K_Cyt
Z_Im_K_Cyt
Z_Shift_L_Gem
Z_Im_L_Gem
Z_Shift_L_Cyt
Z_Im_L_Cyt

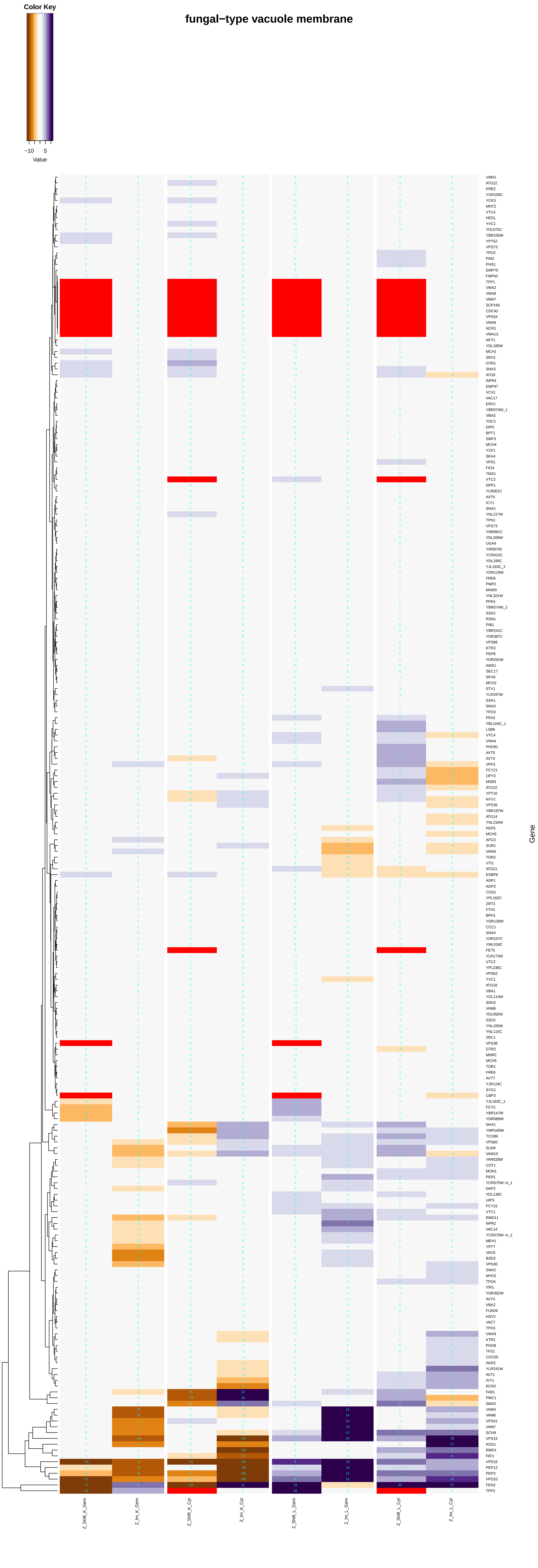
fungal−type vacuole membrane
Color Key
−10
5
Value
Gene
Z_Shift_K_Gem
Z_Im_K_Gem
Z_Shift_K_Cyt
Z_Im_K_Cyt
Z_Shift_L_Gem
Z_Im_L_Gem
Z_Shift_L_Cyt
Z_Im_L_Cyt

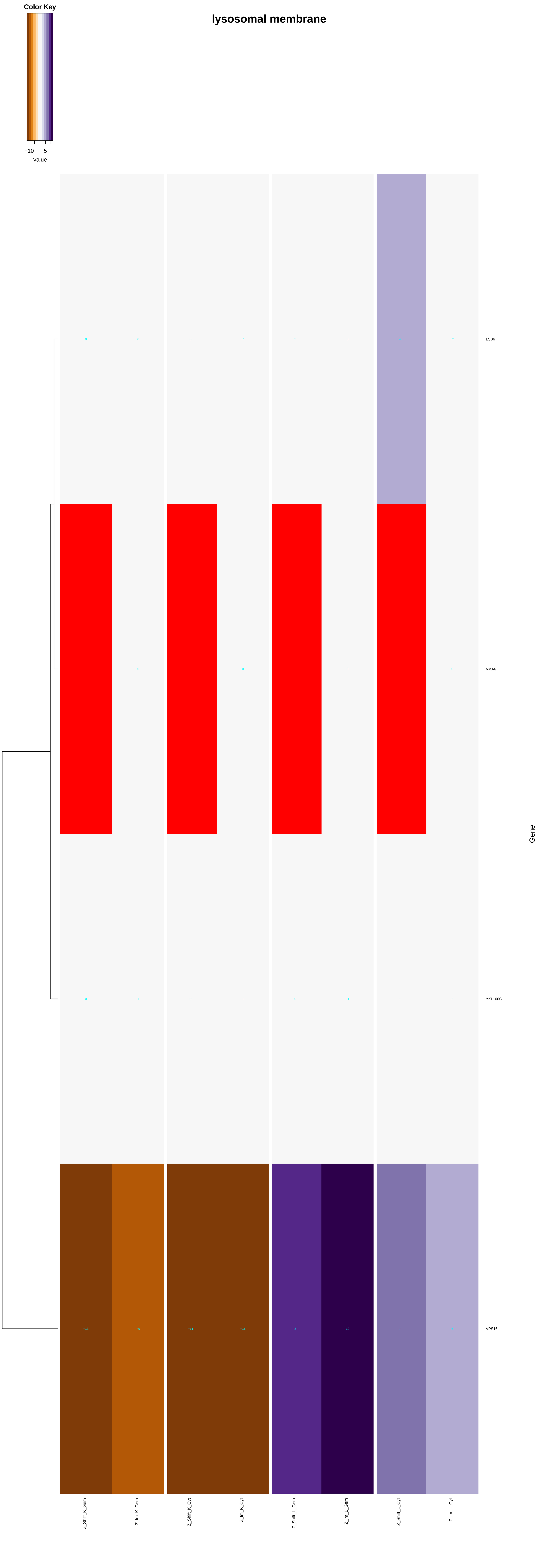

lysosomal membrane
Color Key
−10
5
Value
LSB6
VMA6
YKL100C
VPS16
Gene
Z_Shift_K_Gem
Z_Im_K_Gem
Z_Shift_K_Cyt
Z_Im_K_Cyt
Z_Shift_L_Gem
Z_Im_L_Gem
Z_Shift_L_Cyt
Z_Im_L_Cyt

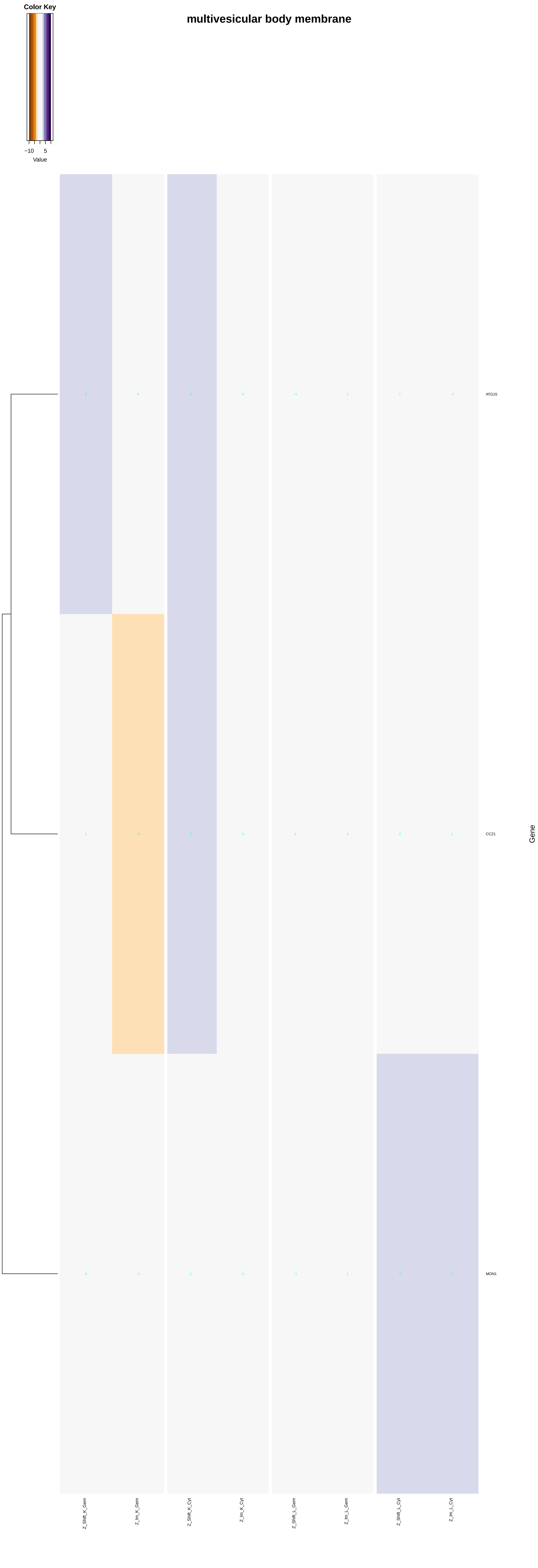
multivesicular body membrane
Color Key
−10
5
Value
ATG15
CCZ1
MON1
Gene
Z_Shift_K_Gem
Z_Im_K_Gem
Z_Shift_K_Cyt
Z_Im_K_Cyt
Z_Shift_L_Gem
Z_Im_L_Gem
Z_Shift_L_Cyt
Z_Im_L_Cyt
